# ADORA2A-driven proline synthesis triggers epigenetic reprogramming in neuroendocrine prostate and lung cancers

**DOI:** 10.1172/JCI168670

**Published:** 2023-12-15

**Authors:** Na Jing, Kai Zhang, Xinyu Chen, Kaiyuan Liu, Jinming Wang, Lingling Xiao, Wentian Zhang, Pengfei Ma, Penghui Xu, Chaping Cheng, Deng Wang, Huifang Zhao, Yuman He, Zhongzhong Ji, Zhixiang Xin, Yujiao Sun, Yingchao Zhang, Wei Bao, Yiming Gong, Liancheng Fan, Yiyi Ji, Guanglei Zhuang, Qi Wang, Baijun Dong, Pengcheng Zhang, Wei Xue, Wei-Qiang Gao, Helen He Zhu

**Affiliations:** 1 State Key Laboratory of Systems Medicine for Cancer, Renji-Med-X Stem Cell Research Center, Department of Urology, Ren Ji Hospital, Shanghai Cancer Institute, School of Medicine and School of Biomedical Engineering, and; 2 Med-X Research Institute, Shanghai Jiao Tong University, Shanghai, China.; 3 Emergency Intensive Care Unit, Shanghai Seventh People’s Hospital of Shanghai University of Traditional Chinese Medicine, Shanghai, China.; 4 Department of Thoracic Surgery, Shanghai Pulmonary Hospital, Tongji University School of Medicine, Shanghai, China.; 5 Department of Obstetrics and Gynecology, Shanghai Cancer Institute, Shanghai Key Laboratory of Gynecologic Oncology, Ren Ji Hospital, Shanghai Jiao Tong University School of Medicine, Shanghai, China.; 6 School of Biomedical Engineering, ShanghaiTech University, Shanghai, China.

**Keywords:** Metabolism, Oncology, Epigenetics, Lung cancer, Prostate cancer

## Abstract

Cell lineage plasticity is one of the major causes for the failure of targeted therapies in various cancers. However, the driver and actionable drug targets in promoting cancer cell lineage plasticity are scarcely identified. Here, we found that a G protein-coupled receptor, ADORA2A, is specifically upregulated during neuroendocrine differentiation, a common form of lineage plasticity in prostate cancer and lung cancer following targeted therapies. Activation of the ADORA2A signaling rewires the proline metabolism via an ERK/MYC/PYCR cascade. Increased proline synthesis promotes deacetylases SIRT6/7-mediated deacetylation of histone H3 at lysine 27 (H3K27), and thereby biases a global transcriptional output toward a neuroendocrine lineage profile. Ablation of *Adora2a* in genetically engineered mouse models inhibits the development and progression of neuroendocrine prostate and lung cancers, and, intriguingly, prevents the adenocarcinoma-to-neuroendocrine phenotypic transition. Importantly, pharmacological blockade of ADORA2A profoundly represses neuroendocrine prostate and lung cancer growth in vivo. Therefore, we believe that ADORA2A can be used as a promising therapeutic target to govern the epigenetic reprogramming in neuroendocrine malignancies.

## Graphical abstract

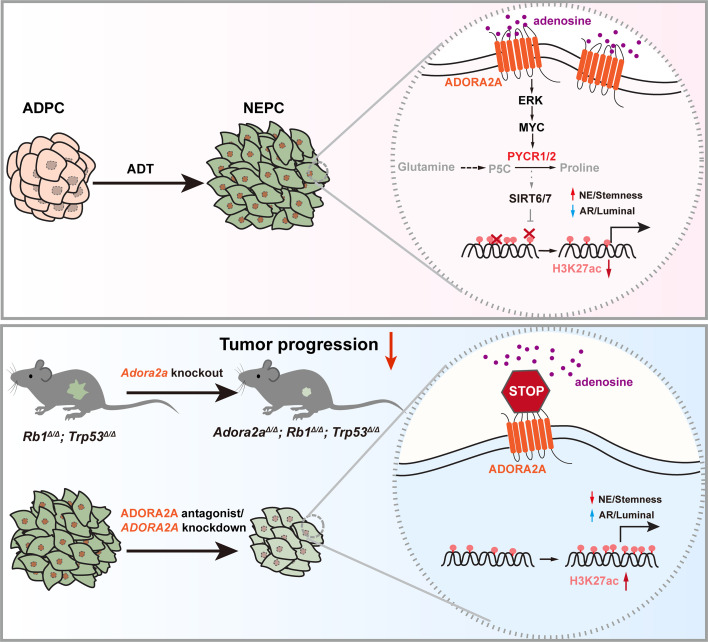

## Introduction

Lineage plasticity is often exploited by cancer cells to acquire therapeutic resistance (1). Lineage transition from adenocarcinoma (AD) to aggressive neuroendocrine (NE) derivatives is a common type of cancer cell plasticity in androgen deprivation therapy–treated (ADT-treated) prostate AD (ADPC) and epidermal growth factor receptor (EGFR) inhibitor–treated lung AD (LUAD) (2). Treatment-induced NE prostate cancer (NEPC) and lung cancer, which display small cell–like carcinoma features and increased expression of neuronal markers, such as synaptophysin (SYP), chromogranin A (CHGA), and neuron-specific enolase (NSE), are highly aggressive and lack effective clinical interventions (3, 4). Therefore, delineating the molecular mechanism by which cancer cells acquire enhanced cell lineage plasticity and identifying actionable drug targets would benefit the development of effective therapeutic strategies for NE cancers.

Metabolic alteration is a hallmark of cancer (5). In contrast to glucose and lipid metabolism, which have been extensively studied in cancer, the metabolism of some specific amino acids has received less attention. Nevertheless, some studies have suggested that proline metabolism is actively involved in tumorigenesis, and that its key enzyme, pyrroline-5-carboxylate reductase 1 (PYCR1) plays an oncogenic role (6, 7). Proline synthesis starts from either glutamine or ornithine, which is converted to the proline precursor pyrroline-5-carboxylate (P5C) by P5C synthetase. Subsequently, 3 P5C reductases, including PYCR1, PYCR2, and PYCR3 catalyze P5C to proline (8). Some metabolites generated from cellular metabolism have been found to influence epigenetic modulations, suggesting a parametabolic role of metabolites in shaping the epigenetic landscape of cancer cells (9). We therefore asked whether proline metabolism is rewired to trigger the AD-to-NE transition in prostate and lung cancer and what the upstream signal is that regulates the proline metabolism.

Adenosine is a purine nucleoside that is generated from ATP by 2 ectonucleotidases, CD39 and CD73, in myeloid-derived suppressor cells (MDSC), endothelial cells, fibroblasts, and other cellular components of the tumor microenvironment (TME) (10, 11). The adenosine receptor A2A (ADORA2A) is a G protein-coupled receptor (GPCR) with a high binding affinity for adenosine (12). Adenosine/ADORA2A signaling has been studied primarily in the CNS during neuronal differentiation and neurogenesis (13, 14). ADORA2A plays a critical role in controlling neuronal excitability, neurotransmitter uptake and release, and synaptic plasticity and stability in the CNS (15, 16). Treatment-induced NE differentiation in cancer cells is proposed to adopt the molecular program of normal neuronal development, based on the observation that the AD-to-NE lineage transition in prostate cancer (PCa) can be evoked by several key neural differentiation transcription factors (TFs) such as ASCL1 (17), ONECUT2 (18), NEUROD1 (19), N-MYC (20, 21), and BRN2 (22). Considering this direction, ADORA2A might be a candidate molecule promoting the AD-to-NE lineage transition of malignancies.

Here, we aim to determine whether and how the adenosine/ADORA2A signaling is involved in the acquisition of the NE transcriptional signature and maintenance of the NE phenotype in NEPC and small cell lung cancer (SCLC). We show that ADORA2A can serve as a promising druggable target that drives a proline metabolic-epigenetic cascade via the ERK/MYC/PYCR axis in NEPC and SCLC, suggesting a broad therapeutic implication of ADORA2A blockade in NE malignancies.

## Results

### ADORA2A is selectively upregulated in NE prostate and lung cancer.

**Figure 1:**
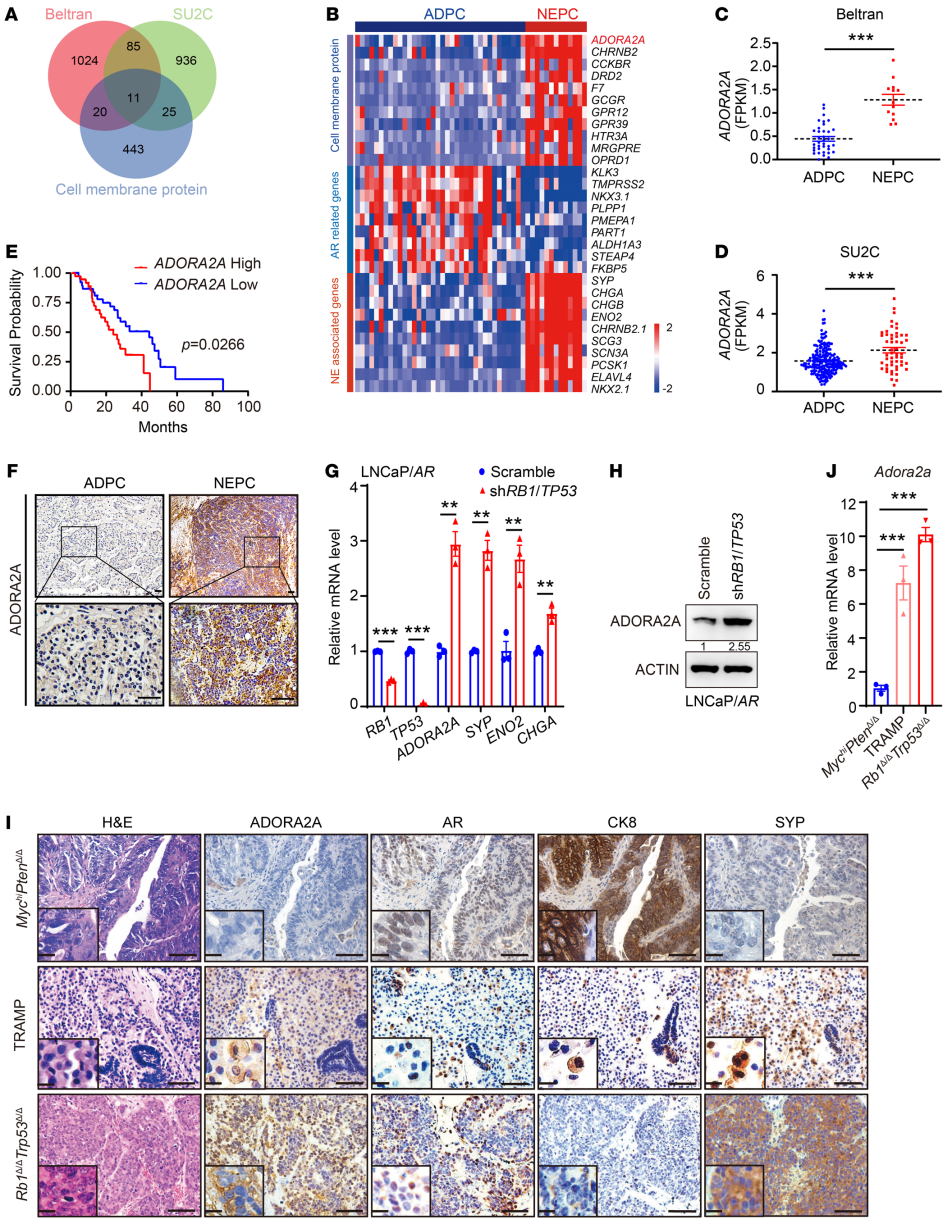
ADORA2A is a selectively upregulated cell membrane protein in NEPC. (**A**) Screening the upregulated cell membrane proteins in NEPC versus ADPC based on reanalysis of Beltran (24) (NEPC, *n* = 13; ADPC, *n* = 36) and SU2C (25) (NEPC, *n* = 52; ADPC, *n* = 214) PCa data sets. (**B**) The heatmap reveals that ADORA2A is a top-ranked cell membrane protein in NEPC versus ADPC based on Beltran PCa data set (24). (**C** and **D**) Quantification of *ADORA2A* mRNA levels in ADPC and NEPC using Beltran (24) (**C**) and SU2C (**D**) PCa data sets (25). (**E**) The Kaplan-Meier survival curves exhibit a significantly shorter survival of patients with a high ADORA2A expression based on SU2C (High, *n* = 38; Low, *n* = 41) PCa data sets (25), cutoff value was 50%. (**F**) Representative IHC showing the ADORA2A levels in ADPC (*n* = 35) and NEPC (*n* = 31) clinical tumor sections. Upper panel scale bar: 200 μm; Lower panel scale bar: 50 μm. (**G**) Representative RT-qPCR shows mRNA levels in LNCaP/*AR*-sh*RB1*/*TP53* and scramble cells (*n* = 3 independent experiments). (**H**) Representative immunoblotting demonstrates an elevated ADORA2A level in LNCaP/*AR*-sh*RB1*/*TP53* compared with scramble cells (*n* = 3 independent experiments). (**I**) H&E showing the histology of *Myc^hi^Pten^Δ/Δ^*, TRAMP, and *Rb1^Δ/Δ^Trp53^Δ/Δ^* prostate tumors from 6-to-8-month-old mice (the left panel). IHC staining demonstrates the expression of ADORA2A, AR, CK8, and SYP in these tumors (scale bar: 100 μm; zoom in area scale bar: 5 μm). (**J**) RT-qPCR showing *Adora2a* levels from organoids from indicated GEMMs (*n* = 3 biological replicates). For statistical analysis, student’s *t* tests were used for **C** and **G**; Mann-Whitney test was utilized for **D**; Log-rank test was employed in **E**; 1-way ANOVA with Dunnett’s posthoc test was applied in **J**. *P < 0.05, **P < 0.01, ***P < 0.001, data are presented as mean ± SEM.

Cell membrane proteins have great potential as druggable targets due to their accessibility to small molecules or blocking antibodies and their important functions in cell signaling transduction (23). To identify promising cell membrane protein targets selectively for NE malignancies, we screened the RNA-Seq data sets from Beltran (24) and the Stand Up To Cancer (SU2C) (25) PCa cohorts, which include ADPC and NEPC clinical samples. We chose the specifically enriched cell membrane protein-encoded genes in NEPC versus ADPC in both data sets (Figure 1A) and found that *ADORA2A* was ranked at the top of the upregulated genes (Figure 1, B–D). ADORA2A is a GPCR that elicits intracellular signaling, such as the AKT and ERK pathways, upon activation by its ligand, adenosine (26, 27). The SU2C PCa data set (25) revealed that patients with high levels of ADORA2A displayed a significantly shorter survival (Figure 1E). To confirm these results, we performed IHC staining on our in-house PCa cohort containing ADPC and NEPC biopsies. The expression of ADORA2A in tumor sections was categorized into low, intermediate, and high levels (Supplemental Figure 1A; supplemental material available online with this article; https://doi.org/10.1172/JCI168670DS1). IHC results showed that human NEPC samples exhibited a higher expression of ADORA2A than ADPC (Figure 1F and Table 1). High levels of ADORA2A were significantly associated with a shorter survival in our own PCa cohort (Supplemental Figure 1B). These data suggest that the elevated ADORA2A level is closely associated with NEPC and predicts a poor clinical outcome in PCa.

Next, we assessed the expression levels of ADORA2A in several PCa cell lines. Immunoblotting data showed that the level of ADORA2A was higher in the NE-like PCa cell line LASCPC-01 (21) than in the other 3 ADPC cell lines including VCaP, LAPC4, and LNCaP (Supplemental Figure 1C). Subsequently, we employed a recently reported LNCaP/*AR* cell line and concomitantly downregulated *RB1* and *TP53* using small hairpin RNAs (shRNA) to generate the LNCaP/*AR*-sh*RB1*/*TP53* derivatives (28) with elevated expression of NE markers including *SYP*, *ENO2*, and *CHGA* (Figure 1, G and H). Real-time quantitative PCR (RT-qPCR) (Figure 1G) and immunoblotting (Figure 1H) results showed that ADORA2A was upregulated in LNCaP/*AR*-sh*RB1*/*TP53* cells compared with their parental LNCaP/*AR* cells at both mRNA and protein levels. In addition, we examined several genetically engineered mouse models (GEMMs) of PCa, including the *Pbsn-Cre4*; *Pten^fl/fl^*; *Hi-Myc* (*Myc^hi^Pten^Δ/Δ^*) (29), TRAMP (30), and *Pbsn-Cre4*; *Rb1^fl/fl^*; *Trp53^fl/fl^* (*Rb1^Δ/Δ^Trp53^Δ/Δ^*) (31), to assess ADORA2A expression patterns in these models. The *Myc^hi^Pten^Δ/Δ^* prostate tumor displayed an ADPC histology of discernible luminal cell morphology with positive androgen receptor (AR) and cytokeratin 8 (CK8) expression (Figure 1I, the upper panel). The TRAMP prostate tumor exhibited a mixed NEPC and ADPC phenotype (Figure 1I, the middle panel). The *Rb1^Δ/Δ^Trp53^Δ/Δ^* tumor showed a prominent small cell–like NE phenotype with increased expression of SYP and weak staining of AR (Figure 1I, the lower panel). Of note, ADORA2A was rarely detected in *Myc^hi^Pten^Δ/Δ^* ADPC tumors but was evidently presented in NE-histological regions with high SYP levels in TRAMP tumors. Strikingly, ADORA2A was robustly expressed in NEPC *Rb1^Δ/Δ^Trp53^Δ/Δ^* tumor sections. RT-qPCR data further demonstrated that *Adora2a* was significantly higher in organoids derived from TRAMP and *Rb1^Δ/Δ^Trp53^Δ/Δ^* NEPC tumors than in those derived from *Myc^hi^Pten^Δ/Δ^* ADPC counterparts (Figure 1J). Immunofluorescence (IF) staining results revealed a strong costaining of ADORA2A and SYP in the NE tumor regions of TRAMP and *Rb1^Δ/Δ^Trp53^Δ/Δ^* mice (Supplemental Figure 1D).

To verify whether the elevation of ADORA2A was a common phenomenon among NE cancers in the lung, we performed IHC staining on a panel of human lung cancer sections including patient samples from 14 patients with LUAD and 19 patients with SCLC. Indeed, SCLC sections showed higher levels of ADORA2A than LUAD (Supplemental Figure 1E and Supplemental Table 1. Further, ADORA2A was expressed more abundantly in 2 SCLC cell lines, NCI-H146 and NCI-H1688, than in LUAD cell lines A549 and SPC-A-1 (Supplemental Figure 1F). Collectively, the experimental results and our analysis of the reported data sets reveal a selective upregulation of ADORA2A in 2 exemplary prostate and lung NE cancers, which is strongly correlated with poor clinical outcomes.

### ADORA2A upregulation following ADT in PCa elicits NE lineage signature.

**Figure 2:**
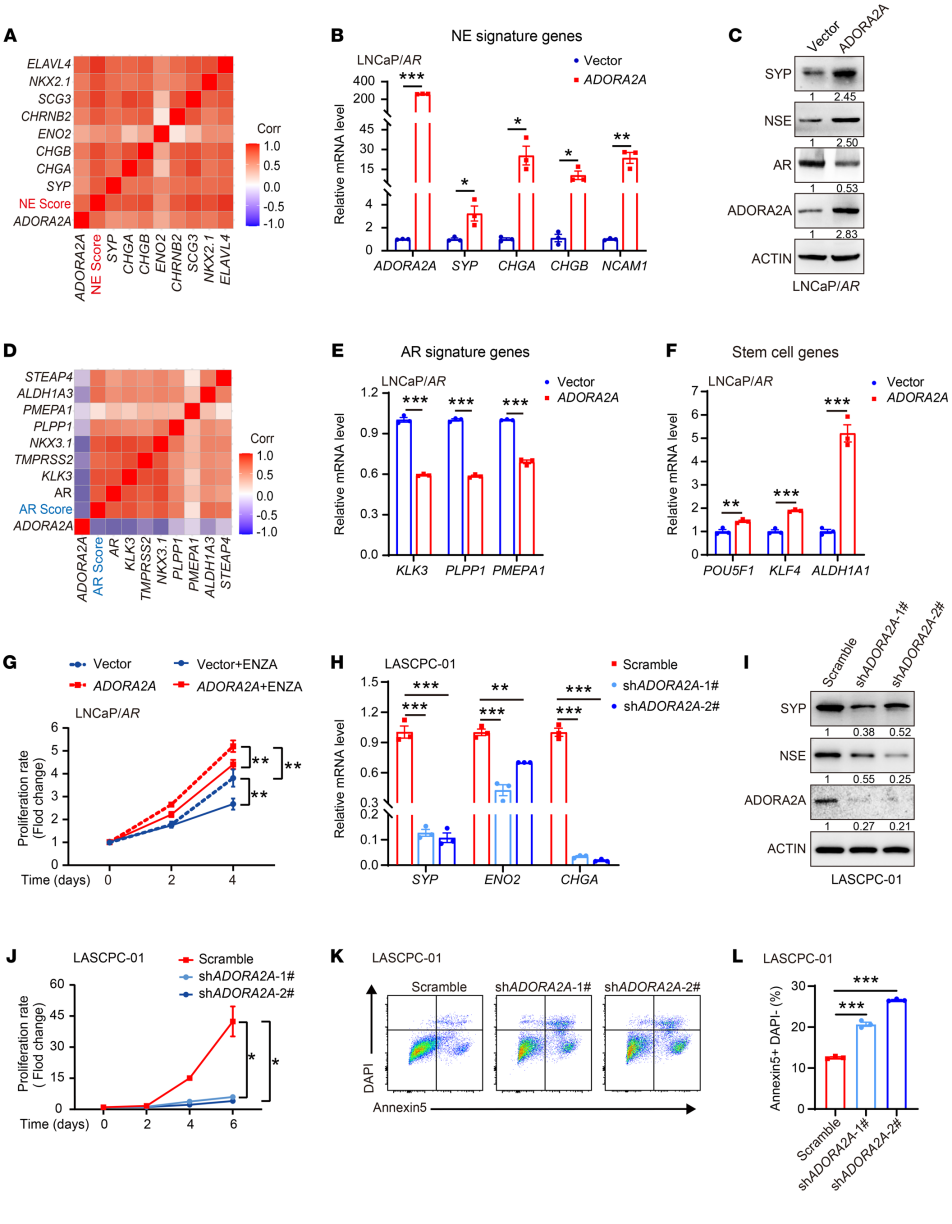
ADORA2A promotes lineage plasticity and resistance to ADT in PCa cells. (**A**) Correlation analysis demonstrates a strong positive association between *ADORA2A* mRNA levels and NE-lineage gene signatures based on the Beltran PCa data set (24). (**B**) RT-qPCR results confirm *ADORA2A*-OE in LNCaP/*AR-ADORA2A* cells and suggest that NE-lineage associated genes are elevated in *ADORA2A*-OE LNCaP/*AR* cells compared with vector cells (*n* = 3). (**C**) Immunoblots of NE-lineage molecules and AR in ADORA2A-OE LNCaP/*AR* cells and vector control cells. (**D**) *ADORA2A* mRNA levels are negatively correlated with expression of AR signaling signature genes based on the analysis of the Beltran PCa data set (24). (**E** and **F**) RT-qPCR analysis of AR signature genes and stem cell marker genes in LNCaP/*AR*-vector and LNCaP/*AR*-*ADORA2A* cells (*n* = 3). (**G**) In vitro cell growth curves of LNCaP/*AR*-*ADORA2A* and LNCaP/*AR*-vector cells cultured in control medium or enzalutamide (ENZA, 15 μM)-containing medium (*n* = 5 biological replicates). (**H** and **I**) RT-qPCR (**H**) and immunoblotting (**I**) results demonstrate NE-lineage genes are decreased in response to the downregulation of *ADORA2A* in LASCPC-01 cells (*n* = 3). (**J**) Cell growth curves of LASCPC-01-scramble cells and LASCPC-01-sh*ADORA2A* cells within 6 days (*n* = 4 biological replicates). (**K** and **L**) Flow cytometry analysis (**K**) and quantification (**L**) of the apoptotic cells in LASCPC-01-scramble and LASCPC-01-sh*ADORA2A* cells (*n* = 3 biological replicates). For statistical analysis, student’s *t* test was used for **B**, **E**, and **F**; 1-way ANOVA with Dunnett’s posthoc test was utilized for **H** and **L**; 2-way ANOVA with Turkey’s posthoc test was applied in **G** and **J**. *P < 0.05, **P < 0.01, ***P < 0.001, data are presented as mean ± SEM. RT-qPCR and immunoblotting were repeated in at least 3 independent experiments, with similar results, and representative images are shown.

The specific upregulation of ADORA2A in NEPC and SCLC prompted us to interrogate its role in the AD-to-NE lineage transition. We revisited the Beltran PCa data set (24) and found that *ADORA2A* displayed a positive association with the NE-associated gene signature (Figure 2A). To verify this result, we overexpressed ADORA2A (ADORA2A-OE) in 2 ADPC cell lines, including LNCaP/*AR* (Figure 2B) and LAPC4 cells (Supplemental Figure 2A), and assessed the transcription of NE-associated genes in these cells. NE-lineage molecules such as SYP, CHGA, CHGB, NCAM1, and NSE (encoded by *ENO2*) were upregulated upon ADORA2A-OE in the presence of the adenosine analog CGS21680 (CGS) (Figure 2, B and C, and Supplemental Figure 2, A–D). On the other hand, an inverse correlation between the *ADORA2A* mRNA level and AR signaling signature was observed in the Beltran PCa data set (24) (Figure 2D). Consistently, AR and its targets, including *KLK3*, *PLPP1*, and *PMEPA1* were significantly reduced upon ADORA2A-OE in LNCaP/*AR* and LAPC4 cells (Figure 2, C and E, and Supplemental Figure 2C). RT-qPCR results further showed that ADORA2A-OE significantly upregulated stem cell-related genes, such as *POU5F1*, *KLF4*, and *ALDH1A1* (Figure 2F). Next, we knocked down ADORA2A (ADORA2A-KD) in the NEPC-like cell line LNCaP/*AR*-sh*RB1/TP53* (Supplemental Figure 2E) and assessed the expression levels of NE-associated genes, stem cell–related genes, and AR signaling genes. The levels of NE lineage genes (Supplemental Figure 2, E and F) and stem cell marker genes (Supplemental Figure 2G) were significantly decreased, but AR signaling target genes (Supplemental Figure 2H) were restored upon ADORA2A-KD in LNCaP/*AR*-sh*RB1*/*TP53* cells. These results suggest that ADORA2A promotes lineage plasticity and facilitates PCa cells to acquire an NE-lineage biased phenotype. ADORA2A-OE conferred a growth advantage to LNCaP/*AR* cells in both normal and enzalutamide-containing (ENZA-containing) medium (Figure 2G). This growth advantage was diminished upon the ADORA2A-KD in LNCaP/*AR*-sh*RB1*/*TP53* cells (Supplemental Figure 2I). Similarly, in another NEPC-like cell line LASCPC-01, ADORA2A-KD (Supplemental Figure 2J) not only suppressed NE lineage molecule expression (Figure 2, H and I), but also significantly inhibited cell proliferation (Figure 2J) and promoted cell apoptosis (Figure 2, K and L).

We next sought to determine the mechanism that triggers ADORA2A upregulation in NEPC. Loss of dependency on AR signaling is a key event during NE differentiation (32). Therefore, we examined the effect of AR signaling on ADORA2A expression. Ectopic expression of AR in LNCaP cells (Supplemental Figure 3A) suppressed ADORA2A expression at both the mRNA (Supplemental Figure 3B) and protein levels (Supplemental Figure 3C). Moreover, ADORA2A was decreased in LNCaP/*AR* cells following the treatment of the AR agonist R1881, and this trend was reversed by the AR inhibitor ENZA (Supplemental Figure 3D). Genetic ablation of *AR* in LNCaP cells using the CRISPR/Cas9 method increased ADORA2A expression (Supplemental Figure 3E). To validate whether AR signaling status affects *ADORA2A* transcriptional activity, we incorporated the core fragment of the *ADORA2A* promoter sequence into a luciferase reporter construct and assessed luciferase activity upon AR activation or blockade. Addition of R1881 significantly decreased *ADORA2A* transcriptional activity. This trend was reversed when AR signaling was blocked by ENZA (Supplemental Figure 3F). On the other hand, LNCaP-sg*AR* cells displayed a significantly increased *ADORA2A* transcriptional activity compared to the scrambled sgRNA control cells (Supplemental Figure 3G). In support of these results, an inverse correlation between the staining intensity of ADORA2A and a typical AR target, PSA, was identified in human PCa tumor sections (Supplemental Figure 3, H and I). In summary, our data demonstrate that the transcriptional activity of *ADORA2A* is restrained by AR signaling and released upon AR blockade in PCa.

It has been reported that several neuronal TFs, such as ONECUT2 (18), NEUROD1 (19), N-MYC (20, 21), and ASCL1 (17), facilitate the activation of the NE transcriptional program and promote the lineage transition in PCa. We next asked whether these NE-related TFs were involved in the upregulation of ADORA2A expression. Using the Cistrome Data Browser (cistrome.org/db), we found that among these TFs, only ASCL1 displayed evident binding peaks in the promoter region of *ADORA2A* in PCa cells (Supplemental Figure 3J). Luciferase reporter assay confirmed that ectopic expression of ASCL1 (Supplemental Figure 3K) increased the *ADORA2A* transcriptional activity in PCa cells (Supplemental Figure 3L). Ectopic expression of ASCL1 upregulated ADORA2A expression at both the mRNA and protein levels (Supplemental Figure 3, M and N), suggesting that ASCL1 acts as a transcriptional activator for *ADORA2A*. Collectively, these results suggest that ADORA2A is suppressed by AR signaling and activated by ASCL1 in PCa cells.

### ADORA2A promotes proline synthesis by the upregulation of PYCR.

**Figure 3:**
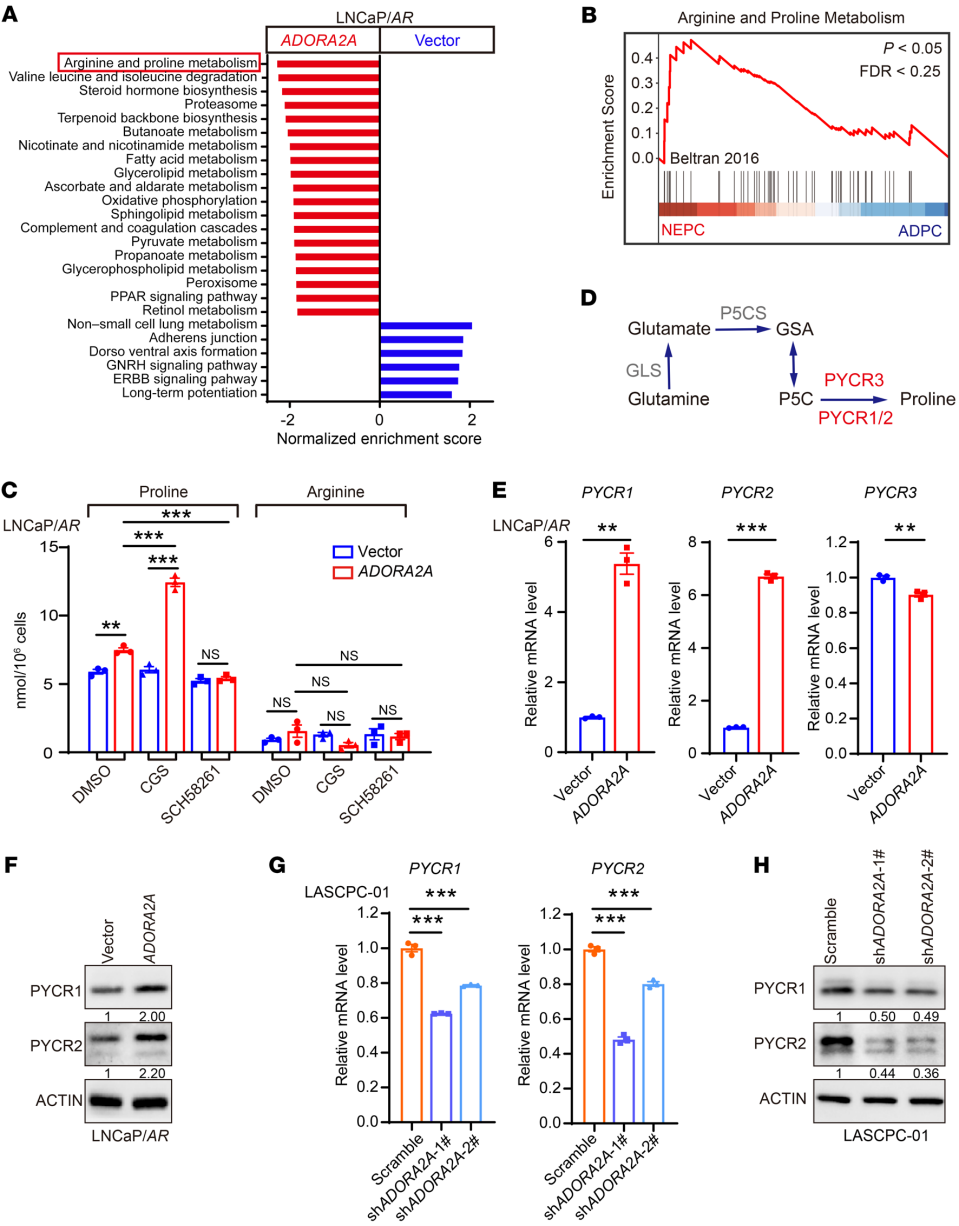
ADORA2A signaling promotes the proline synthesis by upregulating PYCRs. (**A**) GSEA analysis reveals upregulated biological processes and pathways in KEGG enrichment analysis in LNCaP/*AR*-*ADORA2A* versus LNCaP/*AR*-vector cells (*n* = 3 biological replicates per cell line). (**B**) The GSEA plot shows that arginine and proline metabolism-related genes are enriched in NEPC compared with ADPC based on analysis of the Beltran PCa data set (24). (**C**) Mass spectrometry assesses the intracellular amount of proline and arginine in LNCaP/*AR*-vector and LNCaP/*AR-ADORA2A* cells in the absence or in the presence of ADORA2A agonist CGS (100 nM, treated for 48 hours) or antagonist SCH58261 (25 μM, treated for 48 hours) (*n* = 3 biological replicates/group). (**D**) The schematic flowchart displays the proline synthesis and key enzymes. (**E** and **F**) RT-qPCR (**E**) and immunoblotting (**F**) assays reveal the expression levels of *PYCR1*, *PYCR2*, and *PYCR3* in response to ectopic expression of *ADORA2A* in LNCaP/*AR* cells (*n* = 3). (**G**) RT-qPCR data demonstrate decreases of *PYCR1* and *PYCR2* transcription upon the downregulation of *ADORA2A* via shRNA in LASCPC-01 cells (*n* = 3). (**H**) Immunoblotting results display reduced PYCR1 and PYCR2 at the protein level in response to ADORA2A knockdown in LASCPC-01 cells. For statistical analysis, 1-way ANOVA with Turkey’s posthoc test and Kruskal-Wallis test with Dunnett’s posthoc test was utilized for **C**; student’s *t* test was used for **E**; 1-way ANOVA with Dunnett’s posthoc test was applied in **G**.**P < 0.01, ***P < 0.001, data are presented as mean ± SEM. RT-qPCR and immunoblotting were repeated in 3 independent experiments, with similar results, and representative images are shown.

To understand the mechanism by which ADORA2A facilitates the cell lineage plasticity and induces the AD-to-NE phenotypic transition, we performed RNA-Seq to analyze the transcriptional profile of LNCaP/*AR*-vector versus LNCaP/*AR*-*ADORA2A* cells in the presence of CGS. Gene set enrichment analysis (GSEA) of RNA-Seq data showed that the arginine and proline metabolism pathway was among the top upregulated metabolic pathways in LNCaP/*AR*-*ADORA2A* cells compared with vector cells (Figure 3A and Supplemental Figure 4A). In the experimental comparison set of vehicle-treated versus CGS-stimulated LNCaP/*AR*-*ADORA2A* cells, arginine and proline metabolism also ranked first on the list of upregulated metabolic signaling in CGS-stimulated ADORA2A-OE LNCaP/*AR* cells compared with vehicle-treated counterparts (Supplemental Figure 4B). Consistently, the GSEA analysis of the Beltran PCa data set (24) showed that NEPC samples were also featured with a stronger arginine and proline metabolism hallmark gene signature compared with ADPC counterparts (Figure 3B and Supplemental Figure 4C). Based on these findings, we sought to determine the intracellular levels of arginine and proline using mass spectrometry. Indeed, LNCaP/*AR*-*ADORA2A* cells displayed a higher level of proline than LNCaP/*AR*-vector cells (Figure 3C). Notably, in LNCaP/*AR*-*ADORA2A* cells, the level of proline was significantly increased upon the treatment of the ADORA2A agonist CGS and was decreased by the ADORA2A antagonist SCH58261 (SCH) (Figure 3C). Due to the lack of ADORA2A in LNCaP/*AR*-vector cells, the amounts of intracellular proline were not altered by either CGS or SCH treatment (Figure 3C). However, the levels of arginine in LNCaP/*AR*-*ADORA2A* cells were not influenced by ADORA2A signaling status, indicating a specific effect of ADORA2A signaling on the induction of proline synthesis. These results suggest that the intracellular proline level in PCa cells is regulated by the ADORA2A signaling.

To decipher the mechanism of the increased amount of proline upon the activation of ADORA2A signaling, we compared the key genes of enzymes that catalyze the proline synthesis, including *PYCR1*, *PYCR2*, and *PYCR3* (Figure 3D), between NEPC and ADPC using the Beltran PCa data set (24). Interestingly, *PYCR2*, which controls the final step of proline synthesis, was identified as a top-ranking proline synthesis gene in NEPC compared with ADPC (Supplemental Figure 4C). To explore whether other *PYCRs* could be upregulated by ADORA2A signaling, we conducted RT-qPCR assay and found that only *PYCR1* and *PYCR2* were significantly increased in LNCaP/*AR*-*ADORA2A* cells compared with control cells maintained in CGS-containing medium (Figure 3E). Consistently, immunoblotting results confirmed that the levels of PYCR1 and PYCR2 were higher in LNCaP/*AR*-*ADORA2A* than those in LNCaP/*AR*-vector cells in the presence of CGS (Figure 3F). On the other hand, ADORA2A-KD in LASCPC-01 cells led to reductions of PYCR1 and PYCR2 at both mRNA (Figure 3G) and protein (Figure 3H) levels. To assess whether PYCR1/2 are required for cell proliferation, we downregulated *PYCR1* and *PYCR2* in LNCaP/*AR-ADORA2A* cells and performed cell cycle analysis. As shown in Supplemental Figure 4D, LNCaP/*AR*-*ADORA2A* cells showed an inhibited cell cycle progression in response to the downregulation of either *PYCR1* or *PYCR2*. Furthermore, we asked whether the increased amount of proline could in turn affect cell proliferation and drug resistance of PCa cells. To this end, we compared the growth of LNCaP/*AR*-vector and LNCaP/*AR*-*ADORA2A* cells in both proline-containing and proline-free cell culture conditions. Interestingly, the absence or presence of proline had little effect on either cell proliferation or sensitivity to enzalutamide of LNCaP/*AR*-*ADORA2A* and vector cells (Supplemental Figure 4E). Therefore, it is likely that the proline synthesis process, rather than proline per se*,* plays a role in the AD-to-NE transition in PCa cells. In summary, our data demonstrate that the activation of ADORA2A signaling promotes proline production by upregulation of PYCR1/2 in PCa cells.

### The ADORA2A signaling induces a phenotypic switch from the AD to NE lineage via the ERK/MYC/PYCR axis.

**Figure 4:**
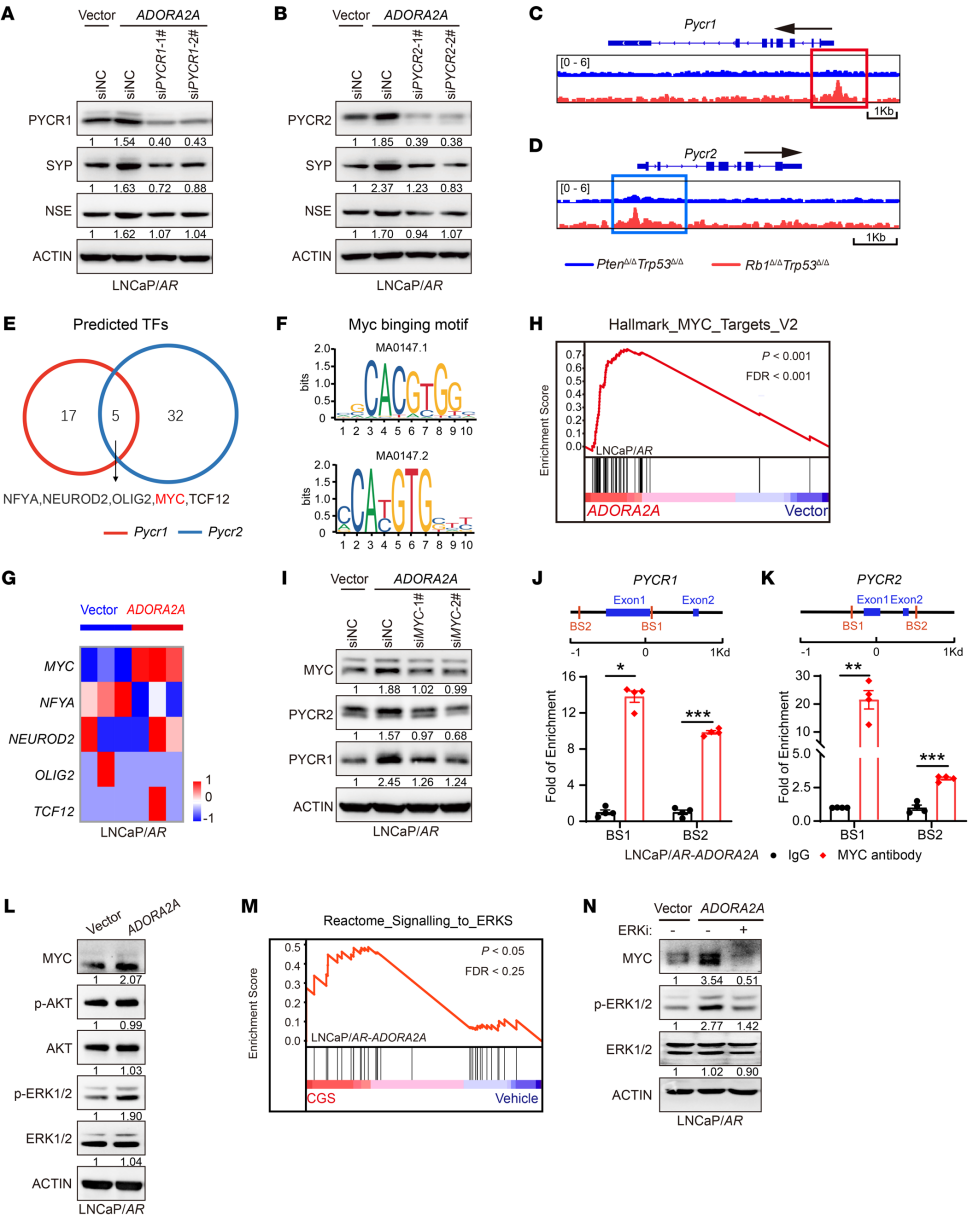
ADORA2A facilitates the acquisition of NE-lineage signature in PCa cells via an ERK/MYC/PYCR axis. (**A** and **B**) Immunoblotting assays demonstrate reduced SYP and NSE levels in LNCaP/*AR-ADORA2A* cells in the presence of CGS (100 nM, treated for 48 hours) upon siRNA-mediated downregulations of *PYCR1* (**A**) and *PYCR2* (**B**). (**C** and **D**) ATAC-Seq show that *Rb1*^Δ***/***Δ^*Trp53*^Δ***/***Δ^ GEMM organoids display more accessible chromatin in the promoter region of *Pycr1* (**C**) and *Pycr2* (**D**) than *Pten*^Δ***/***Δ^*Trp53*^Δ***/***Δ^ counterparts (*n* = 2 biological replicates per cell lines). (**E** and **F**) Motif analysis identifies the binding site of 5 TFs (**E**) on the promoter region of *Pycr1* and *Pycr2* genes using JASPAR. The binding motif of MYC (**F**) on the promoters of *Pycr1* and *Pycr2* are displayed. (**G**) RNA-Seq data of LNCaP/*AR*-vector and LNCaP/*AR-ADORA2A* cells in the presence of CGS reveals that *MYC* is a significantly upregulated transcription factor following the ADORA2A activation. (**H**) The GSEA plot shows that MYC signaling–related genes are enriched in LNCaP/*AR*-*ADORA2A* cells versus LNCaP/*AR*-vector cells. (**I**) Immunoblotting results demonstrate decreased PYCR1 and PYCR2 protein levels upon downregulation of *MYC* via siRNAs in LNCaP/*AR-ADORA2A* cells treated with CGS21680. (**J** and **K**) ChIP-qPCR results show the binding of MYC with the indicated sites of *PYCR1* (**J**) and *PYCR2* (**K**) promoter in LNCaP/*AR-ADORA2A* cells stimulated by CGS (*n* = 4). (**L**) Immunoblotting assay displays upregulated MYC and phospho-ERK1/2 levels in LNCaP/*AR-ADORA2A* compared with LNCaP/*AR-*vector cells in the presence of CGS. (**M**) The GSEA plot reveals that ERK signaling–related genes are enriched in CGS-stimulated versus vehicle-treated ADORA2A-overexpressed LNCaP/*AR* cells. (**N**) Immunoblotting assay reveals a reduced MYC expression level upon the treatment of ERK inhibitor GDC-0994 (10 μM, treated for 48 hours) in LNCaP/*AR-ADORA2A* cells. For statistical analysis, student’s *t* test was used for **J** and **K**. *P < 0.05, **P < 0.01, ***P < 0.001, data are presented as mean ± SEM. For RNA-Seq, *n* = 3 biological replicates/group; immunoblotting was repeated in 3 independent experiments, with similar results, and representative images are shown.

The ADORA2A signaling–induced NE-lineage signature was suppressed upon the knockdown of either *PYCR1* (Figure 4A) or *PYCR2* (Figure 4B), suggesting that the ADORA2A-mediated AD-to-NE lineage transition may rely on PYCR1 and PYCR2. To explore upstream regulators of PYCR1 and PYCR2 in NEPC, we performed ATAC-Seq on PCa organoids from a typical ADPC GEMM *Pbsn-Cre4*; *Pten^fl/fl^*; *Trp53^fl/fl^* (*Pten*^Δ***/***Δ^*Trp53*^Δ***/***Δ^) and the NEPC *Rb1*^Δ***/***Δ^*Trp53*^Δ***/***Δ^ mouse line. As a result, *Rb1*^Δ***/***Δ^*Trp53*^Δ***/***Δ^ organoids displayed a more accessible chromatin state in the promoter regions of the *Pycr1* (Figure 4C) and *Pycr2* (Figure 4D) genes than *Pten*^Δ***/***Δ^*Trp53*^Δ***/***Δ^ organoids. We next examined these activated promoter regions and performed a motif enrichment analysis. By overlapping the binding motif of potential TFs in the accessible region of *Pycr1*/*2* promoters, we identified several TF binding motifs, including NFYA, NEUROD2, OLIG2, MYC, and TCF12, in both *Pycr1* and *Pycr2* promoters, implying that the TFs may occupy these promoter regions and regulate PYCR1/2 expression (Figure 4, E and F). By analyzing our RNA-Seq data, we found that MYC was the only one among these TFs that was selectively upregulated in the LNCaP/*AR-ADORA2A* cells compared with control cells maintained in CGS-containing medium (Figure 4G). Consistently, the GSEA plot showed an enrichment of MYC target genes in LNCaP/*AR*-*ADORA2A* cells versus vector cells (Figure 4H). To verify whether MYC was required for ADORA2A-mediated PYCR1/2 upregulation, we knocked down *MYC* in LNCaP/*AR*-*ADORA2A* cells and found that the ADORA2A signaling–mediated upregulation of PYCR1/2 was abolished compared with control cells (Figure 4I). ChIP-qPCR data further validated the binding sites of MYC on the promoter regions of *PYCR1* (Figure 4J) and *PYCR2* (Figure 4K) genes. Our data indicate that MYC may serve as a key TF of *Pycr1/2* in PCa cells.

We next explored the downstream cascade of ADORA2A signaling leading to the elevation of MYC. Based on previous reports, we assessed 2 classic ADORA2A downstream pathways, including the AKT and ERK signaling (26, 27) in PCa. Interestingly, the ERK pathway, but not the AKT pathway, was notably activated in LNCaP/*AR-ADORA2A* cells in the presence of CGS (Figure 4L). In line with this result, the GSEA plot showed a stronger ERK signaling signature in CGS-stimulated LNCaP/*AR*-*ADORA2A* cells compared with vehicle-treated cells (Figure 4M). On the other hand, blockade of the ERK signaling by a small molecule inhibitor attenuated the ADORA2A signaling-induced MYC upregulation in PCa cells (Figure 4N). Thus, based on published reports, our own experimental results, and bioinformatics analysis, ERK signaling and MYC may serve as key downstream effectors of ADORA2A signaling and lead to the upregulation of PYCR1 and PYCR2 in PCa cells.

### Activated ADORA2A signaling rewires the global histone acetylation status in PCa cells via SIRT6/7.

**Figure 5:**
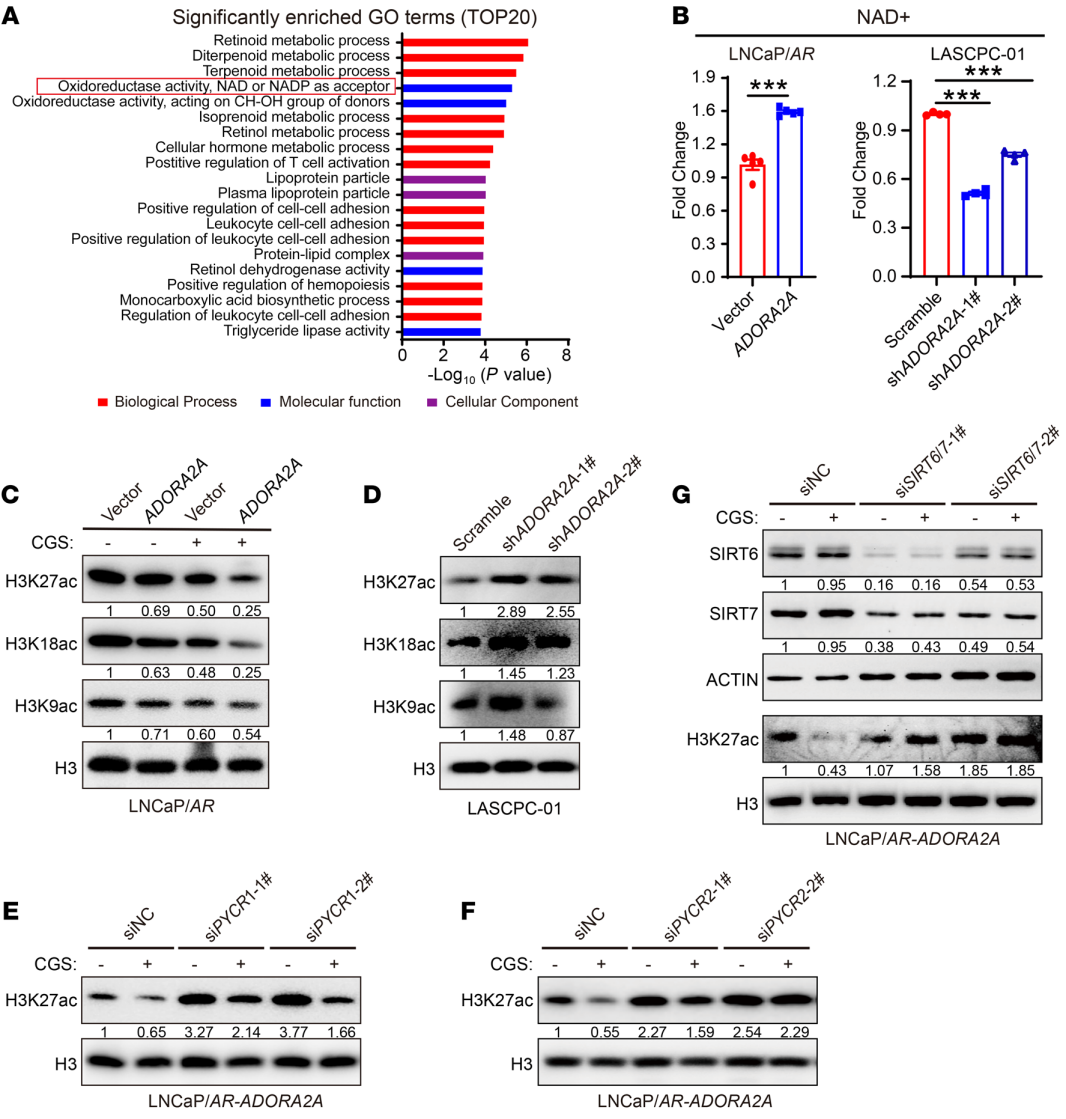
Enhanced proline synthesis reprograms global histone acetylation in PCa cells. (**A**) GO analysis showing the significantly upregulated biological processes, molecular functions, and cellular components in LNCaP/*AR*-*ADORA2A* versus LNCaP/*AR*-vector cells pretreated with CGS. (**B**) Measurement of intracellular amount of NAD^+^ in LNCaP/*AR*-vector and LNCaP/*AR*-*ADORA2A* cells (left, *n* = 5 biological replicates/group), and LASCPC-01-scramble and LASCPC-01-sh*ADORA2A* cells (right, *n* = 4 biological replicates/group). (**C** and **D**) Immunoblotting assay shows H3K9ac, H3K18ac, and H3K27ac levels of LNCaP/*AR*-vector and LNCaP/*AR*-*ADORA2A* cells,cultured in CGS-containing medium (**C**), and in LASCPC-01-scramble and LASCPC-01-sh*ADORA2A* cells (**D**). (**E** and **F**) Immunoblotting assay exhibits that downregulation of *PYCR1* (**E**) and *PYCR2* (**F**) in LNCaP/*AR*-*ADORA2A* cells restores the decreased levels of H3K27ac in both control medium and CGS-containing medium. (**G**) Immunoblotting results demonstrate that the reduced H3K27ac levels are rescued by combinatory knockdown of SIRT6/7 in LNCaP/*AR-ADORA2A* cells in the presence of CGS. For statistical analysis, student’s *t* test was used for **B**, left panel, and 1-way ANOVA with Dunnett’s posthoc test was employed in **B**, right panel. ***P < 0.001, data are presented as mean ± SEM. Immunoblotting experiments were repeated at least 3 times and representative images are shown.

Since the increased amount of proline did not affect PCa cell proliferation and sensitivity toward ENZA (Supplemental Figure 4E), we proposed that the intermediate metabolite generated during proline synthesis may facilitate the AD-to-NE transition. In the final step of the proline synthesis, NADH is converted to NAD^+^ by PYCRs along with the production of proline. Interestingly, gene ontology (GO) analysis of RNA-Seq data revealed that the “oxidoreductase activity, NAD^+^, NADP^+^ as acceptor” is one of the most prominent molecular functions in LNCaP/*AR-ADORA2A* versus LNCaP/*AR-*vector cells in the presence of CGS (Figure 5A) and in the comparison set of CGS-stimulated versus vehicle-treated LNCaP/*AR*-*ADORA2A* cells (Supplemental Figure 5A). Next, we detected intracellular NAD^+^ content and found that LNCaP/*AR*-*ADORA2A* cells displayed significantly higher levels of NAD^+^ than control cells maintained in CGS-containing medium (Figure 5B, left). Consistently, NAD^+^ levels were significantly decreased upon ADORA2A-KD in LASCPC-01 cells (Figure 5B, right). The NAD^+^ is known to be a key cofactor for the sirtuin family of histone deacetylases (SIRTs) (33). We asked whether ADORA2A signaling upregulation in PCa cells would modulate the landscape of histone deacetylation. ADORA2A-OE suppressed the levels of histone acetylation including H3K9ac, H3K18ac, and H3K27ac in LNCaP/*AR* cells (Figure 5C). Moreover, ADORA2A-KD in LASCPC-01 cells led to an increase of these histone acetylation modifications (Figure 5D). Among them, H3K27ac, a well-defined and most extensively studied mark of enhancers for activated gene transcription, showed the most evident change when ADORA2A was overexpressed (Figure 5C) or downregulated (Figure 5D). We then examined whether the altered modification of H3K27ac elicited by the activated ADORA2A signaling was dependent on PYCR1/2. Knockdown of either *PYCR1* (Figure 5E) or *PYCR2* (Figure 5F) compromised the decrease of H3K27ac levels in LNCaP/*AR*-*ADORA2A* cells upon ADORA2A activation by CGS. When *PYCR1* and *PYCR2* were knocked down simultaneously, the trend toward an increased H3K27ac level was more pronounced than when *PYCR1* or *PYCR2* was downregulated individually (Supplemental Figure 5B), suggesting a redundant function between PYCR1 and PYCR2. Together, these data demonstrate that ADORA2A-triggered PYCR1/2 upregulation reprograms the histone deacetylation landscape in PCa.

Among the SIRT family deacetylases, SIRT1, SIRT6, and SIRT7 are 3 nuclear localized deacetylases that have been reported to catalyze histone deacetylation at H3K27ac (34). Therefore, we focused on these nuclear SIRTs for further investigation to determine which SIRT was primarily responsible for the H3K27ac alteration downstream of ADORA2A. *SIRT1* (Supplemental Figure 5C), *SIRT6* (Supplemental Figure 5D), and *SIRT7* (Supplemental Figure 5E) were individually knocked down in LNCaP/*AR*-*ADORA2A* cells. The changes in H3K27ac levels upon ADORA2A activation by CGS were moderately rescued when *SIRT6* and *SIRT7* were downregulated individually. These results prompted us to ask whether a combinatorial role between SIRTs was involved. When we simultaneously downregulated *SIRT6* and *SIRT7* in LNCaP/*AR*-*ADORA2A* cells, the decreased H3K27ac levels upon ADORA2A signaling activation were restored (Figure 5G), suggesting that the decreased H3K27ac status triggered by the activated ADORA2A was dependent on the combinatorial role of SIRT6 and SIRT7.

**Figure 6:**
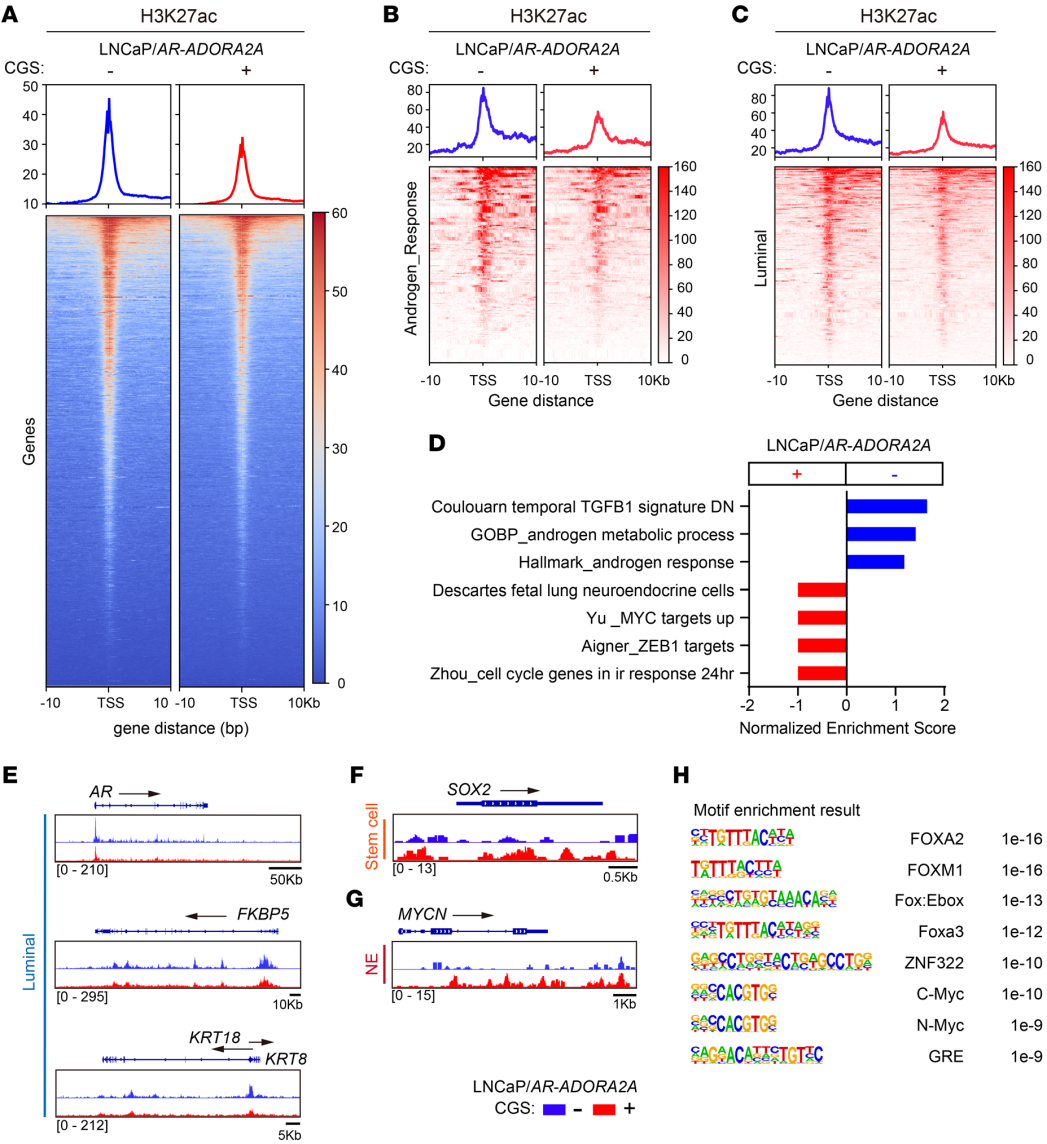
Activation of ADORA2A signaling confers an NE-lineage biased transcription profile to PCa cells. (**A**) The cut & tag data show a repressed H3K27ac level in LNCaP/*AR*-*ADORA2A* cells upon the stimulation of CGS (*n* = 2 independent experiments). (**B** and **C**) Cut & tag results indicate that the H3K27ac mark of androgen responsive genes (**B**) and luminal signature genes (**C**) is decreased in LNCaP/*AR*-*ADORA2A* cells upon CGS stimulation. (**D**) GSEA analysis displays the upregulated hallmarks in LNCaP/*AR*-*ADORA2A* cells upon CGS treatment based on the analysis of differential calling peaks of H3K27ac cut & tag experiments. (**E**) Cut & tag data demonstrate that luminal cell marker genes including *AR*, *FKBP5*, *KRT8,* and *KRT18* promoters contain less H3K27ac marks in LNCaP/*AR*-*ADORA2A* cells in the presence of CGS. (**F** and **G**) Cut & tag results show that stem cell marker gene *SOX2* (**F**) and NE-transcription factor gene *MYCN* (**G**) display increased H3K27ac modifications in LNCaP/*AR*-*ADORA2A* cells upon stimulation. (**H**) Motif analysis exhibits the most enriched transcription factor binding sites on H3K27ac peaks in LNCaP/*AR*-*ADORA2A* cells upon CGS stimulation.

Since H3K27ac is a well-characterized marker of transcriptional activation, we next performed the cut & tag experiments by immunoprecipitation of H3K27ac in LNCaP/*AR-ADORA2A* cells in the absence or presence of CGS to explore the impact of ADORA2A-induced H3K27ac alteration. The overall H3K27ac signal was decreased when ADORA2A signaling was activated (Figure 6A). We next carefully examined the genes that located at the affected H3K27ac sites. As shown in Figure 6, B–D, LNCaP/*AR*-*ADORA2A* cells exhibited reduced H3K27ac levels in androgen-responsive genes (Figure 6B) and luminal cell-associated genes (Figure 6C), but increased H3K27ac signals in NE genes and *MYC* targets (Figure 6D) upon CGS stimulation. In particular, the luminal cell marker genes including *AR*, *FKBP5*, and *KRT8/18* displayed decreased H3K27ac marks in CGS-stimulated LNCaP/*AR*-*ADORA2A* cells (Figure 6E). In contrast, the stem cell marker gene *SOX2* (Figure 6F), and the neuronal differentiation transcription factor *MYCN* (Figure 6G) exhibited more H3K27ac marks in LNCaP/*AR*-*ADORA2A* cells when ADORA2A signaling was stimulated by CGS. Interestingly, motif analysis identified several binding sites of key NE lineage TFs, including FOXA2, and N-MYC, on these H3K27ac peaks of CGS-stimulated LNCaP/*AR-ADORA2A* cells (Figure 6H).

We also conducted H3K27ac cut & tag experiments in ADORA2A-KD and control LASCPC-01 cells and found an overall increased H3K27ac signal upon ADORA2A downregulation (Supplemental Figure 6A). Compared with control cells, H3K27ac peaks in androgen responsive genes (Supplemental Figure 6B) and luminal signature genes (Supplemental Figure 6C) were evidently increased in LASCPC-01-sh*ADORA2A* cells. In particular, the typical AR target genes *FKBP5* and *PSCA* (Supplemental Figure 6D) showed increased H3K27ac marks upon ADORA2A knockdown. H3K27ac marks in the stem cell marker gene *POU5F1* (Supplemental Figure 6E), and the neuronal TF *POU3F2* were suppressed (Supplemental Figure 6F). Collectively, these results suggest that the ADORA2A signaling can affect global H3K27ac modification and alter gene transcriptional output with a decreased luminal signature and an increased NE lineage profile, thereby driving the AD-to-NE lineage transition.

### Genetic ablation of Adora2a in NE prostate and lung cancer mouse models suppresses tumor growth and progression.

**Figure 7:**
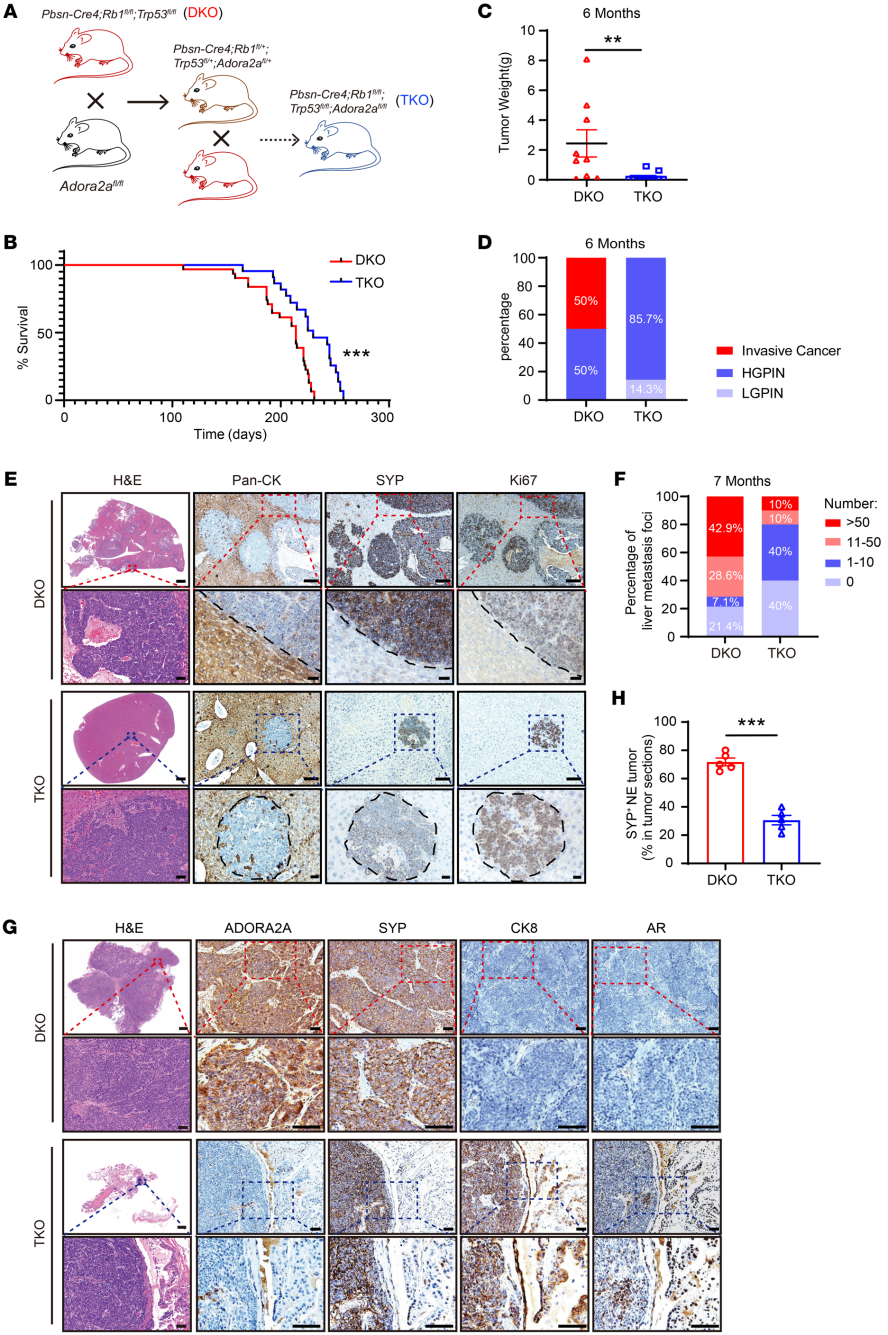
Genetic ablation of *Adora2a* suppresses NEPC development and metastasis. (**A**) A schematic illustrating the generation of *Pbsn-Cre4*; *Rb1^fl/fl^*; *Trp53^fl/fl^*; *Adora2a^fl/fl^* (TKO) GEMMs. (**B**) Kaplan-Meier survival curves indicate a prolonged survival in TKO (*n* = 22) versus DKO (*n* = 31) mice. (**C**) Quantification of tumor weight of TKO (*n* = 9) and DKO (*n* = 11) mice at 6 months of age. (**D**) LGPIN, HGPIN, and invasive cancer on TKO and DKO tumors are quantified. Prostate tumors were collected from DKO (*n* = 8) and TKO (*n* = 7) mice at 6 months of age. (**E**) H&E images display the overall metastatic status in the liver of DKO and TKO mice; scale bar: 1 mm; zoom image scale bar: 50 μm. IHC of Pan-CK outlines the boundary of normal hepatocytes and tumor in the liver. SYP and Ki67 indicate metastatic tumor cells that originate from the prostate; scale bar: 100 μm; zoom image scale bar: 30 μm. (**F**) Quantification of metastatic foci number in DKO (*n* = 14) and TKO (*n*= 10) livers at 7 months old. (**G**) H&E staining demonstrate the whole section of DKO and TKO prostate tumors; scale bar: 1 mm; zoom image scale bar: 50 μm. IHC confirms the absence of ADORA2A in TKO tumors. The NE-lineage marker SYP and luminal cell markers CK8 and AR were stained in DKO and TKO tumors; scale bar: 50 μm; zoom image scale bar: 100 μm. (**H**) The proportion of SYP^+^ NE tumors in DKO (*n* = 5) and TKO (*n* = 5) mice. For statistical analysis, log-rank test was employed in **B**; Mann-Whitney test was utilized for **C**; student’s *t* test was used for **H**. **P < 0.01, ***P < 0.001, data are presented as means ± SEM.

To determine the role of *Adora2a* in NEPC development and progression in vivo, we generated a triple gene–depleted GEMM *Pbsn-Cre4*; *Adora2a^fl/fl^*; *Rb1^fl/fl^*; *p53^fl/fl^* (referred to as TKO) by crossing *Adroa2a^fl/fl^* mice with a previously described *Pbsn-Cre4*; *Rb1^fl/fl^*; *Trp53^fl/fl^* (referred to as DKO) NEPC line (Figure 7A). Ablation of *Adora2a* in prostate epithelial cells significantly prolonged the lifespan of DKO mice (Figure 7B). The tumor weight of TKO mice was also lower than that of DKO mice at 6 months of age (Figure 7C). We further examined the histopathology of prostate tissues from these 2 mouse strains. The histologic presentations were categorized into low-grade prostatic intraepithelial neoplasia (LGPIN), high-grade PIN (HGPIN), and invasive cancer (35). LGPIN was featured with disorganized hyperplastic glands (36, 37). In HGPIN, intraluminal papillary projections and intraductal intruding with a highly proliferative index were detected (3, 31). When HGPIN progressed to invasive cancer, tumor cells disrupted the basement membrane of the gland and invaded the surrounding stroma (38). As shown in Figure 7D, DKO tumors from 6-month-old mice exhibited 50% invasive cancer and 50% HGPIN. In contrast, TKO tumors mainly exhibited 85.7% HGPIN and 14.3% LGPIN with no invasive cancers.

**Figure 8:**
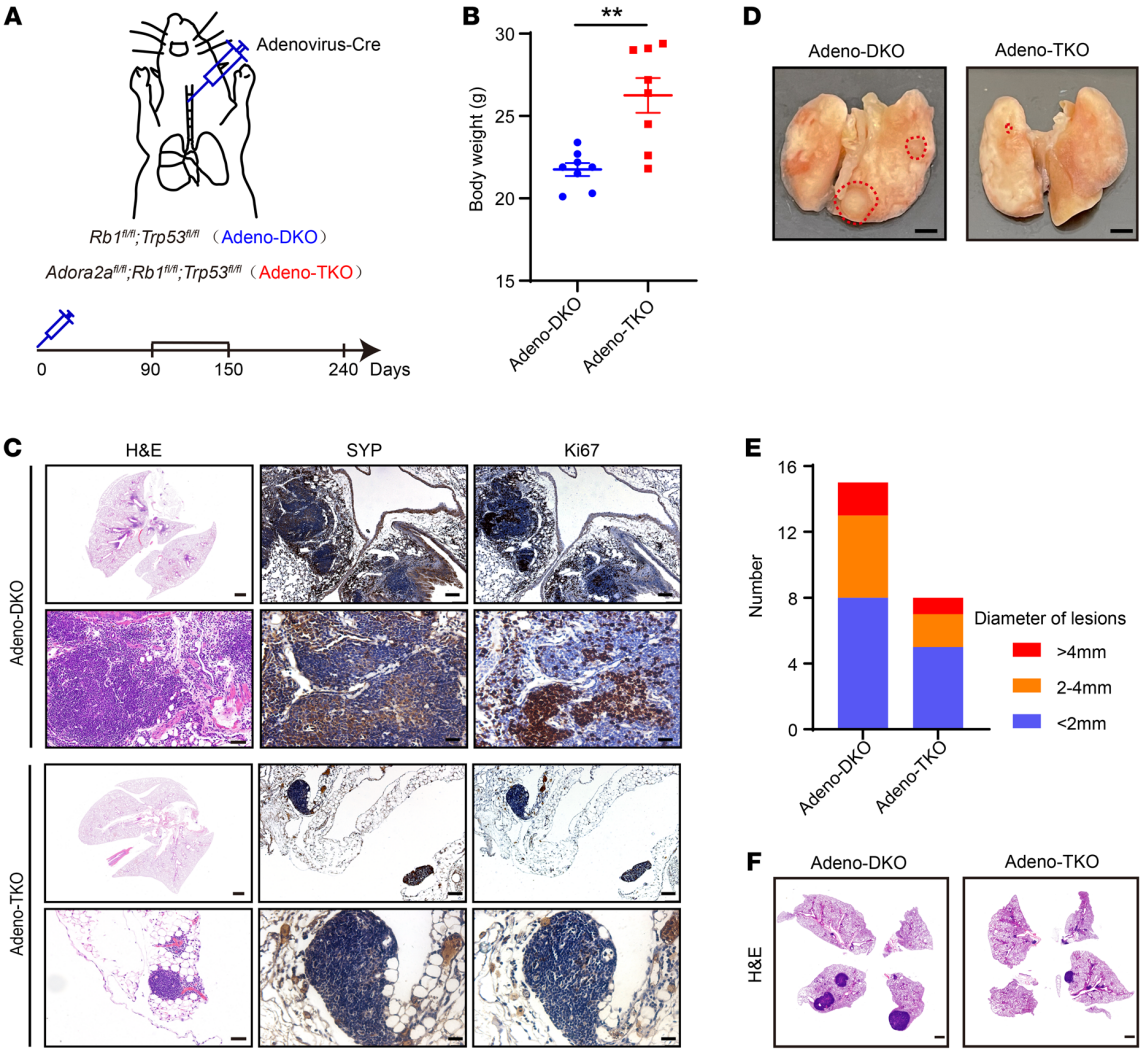
Deletion of *Adora2a* suppresses the tumor development in a NE lung cancer model. (**A**) A schematic showing the method of generation of a NE lung cancer model. Briefly, the Cre-expressing adenovirus (Adeno-Cre) were intratracheally injected into the *Rb1^fl/fl^*; *Trp53^fl/fl^* and *Rb1^fl/fl^*; *Trp53^fl/fl^*; *Adora2a^fl/fl^* mice to establish the Adeno-DKO and Adeno-TKO lung cancer mouse models. (**B**) Body weight of Adeno-DKO (*n* = 8) and Adeno-TKO (*n* = 8) mice at early stage of 90 days after Adeno-Cre injection. (**C**) The H&E staining displays overall tumor formation of Adeno-DKO and Adeno-TKO mice in the lung at early stage; scale bar: 1 mm; zoom image scale bar: 50 μm. IHC images demonstrate the expression of SYP and Ki67 in the lung tumor of Adeno-DKO and Adeno-TKO mice, scale bar = 100 μm; zoom image scale bar: 30 μm. (**D**) The lung tissues of Adeno-DKO and Adeno-TKO mice at late stage of 240 days after Adeno-Cre administration are shown. Dotted red lines indicate the tumor area on the lung tissue. Scale bar: 2mm. (**E**) Quantification of lung tumor lesions with distinct diameters in Adeno-DKO (*n* = 4) and Adeno-TKO (*n* = 3) mice at late stage of 240 days after Adeno-Cre injection. (**F**) H&E images exhibit tumor formations in the lung of Adeno-DKO and Adeno-TKO mice at late stage of 240 days after Adeno-Cre injection. Scale bar: 1 mm. For statistical analysis, student’s *t* test was used for **B**. **P < 0.01, data are presented as mean ± SEM.

Consistent with our previous findings (39), DKO mice developed pronounced liver metastasis at 7 months of age; IHC staining clearly showed the absence of pan-CK, highly expressed SYP, and Ki67 in the liver metastatic foci of DKO mice (Figure 7E), suggesting that they were NEPC cells derived from the prostate. Notably, TKO mice exhibited reduced liver metastasis compared with DKO mice (Figure 7F). The GEMM data strongly support an essential role of *Adora2a* in NEPC development and metastasis in vivo. Interestingly, in contrast to the prominent NE tumor phenotype in DKO prostates, TKO prostates displayed an evident expression of luminal cell markers CK8 and AR and a downregulation of the NE marker SYP (Figure 7, G and H), supporting a function of ADORA2A in modulating PCa cellular plasticity. To further explore the role of ADORA2A in SCLC, we infected the *Rb1^fl/fl^*; *Trp53^fl/fl^* and *Adora2a^fl/fl^*; *Rb1^fl/fl^*; *Trp53^fl/fl^* mice with the Cre recombinase–expressing adenovirus (Adeno-Cre) through intratracheal injection (Figure 8A) to generate Adeno-DKO and Adeno-TKO lung tumors. The Adeno-DKO mice developed SYP^+^ NE-like lung cancer at approximately 90 days and eventually died at around 250 days after the administration of Adeno-Cre viruses. Therefore, we chose 2 time points including 90 days as early stage and 240 days as late stage after Adeno-Cre administration. The body weight of Adeno-TKO mice was significantly higher than that of Adeno-DKO mice at the late stage (Figure 8B), indicating that *Adora2a* ablation improved the overall health status of Adeno-DKO animals. At 90 days, Adeno-DKO mice began to develop evident lung tumor foci locally around the trachea and bronchus, while Adeno-TKO mice only exhibited rare tumor formation with a much smaller tumor size at this time point. As shown in Figure 8C, the Adeno-DKO mice displayed more patches of SYP^+^Ki67^+^ tumors in the lung. In comparison, the Adeno-TKO lung showed much less tumor formation, with weaker SYP signals and decreased Ki67 index throughout the whole tissue section. At 240 days, we found that Adeno-TKO lung tissues had fewer number of tumors (Figure 8, D and E). H&E staining results revealed that Adeno-DKO mice developed much larger tumor areas throughout the lung tissue compared with their Adeno-TKO counterparts (Figure 8F). These data suggest that *Adora2a* ablation suppressed the NE tumor development in vivo in both prostate and lung cancer.

### Pharmacological inhibition of ADORA2A restrains NE prostate and lung tumor growth in vivo.

**Figure 9:**
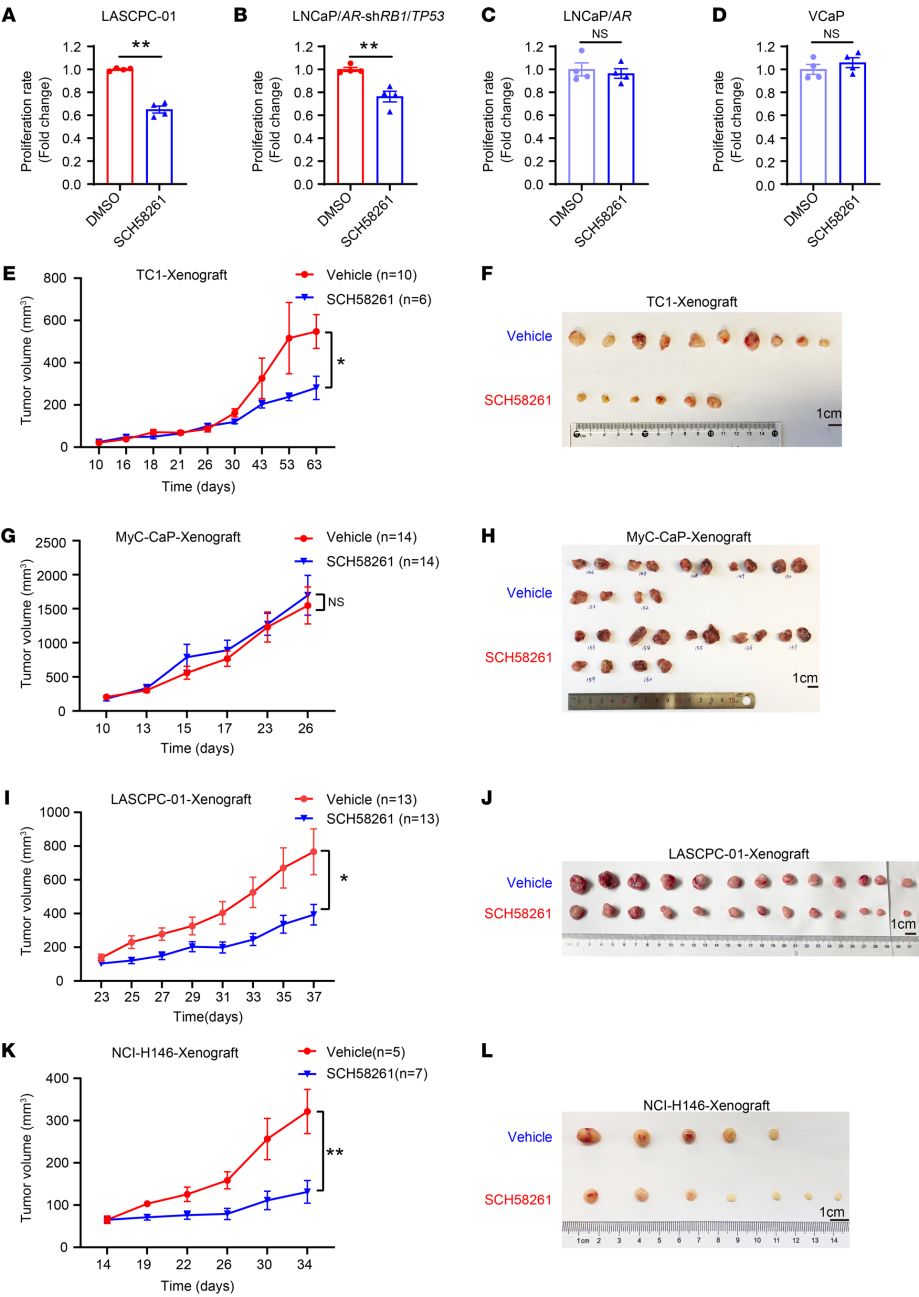
Pharmacological inhibition of ADORA2A restrains NE tumor growth in vitro and in vivo. (**A** and **B**) The Cell Titer Glo assay shows that the ADORA2A antagonist SCH58261 restrains the proliferation of LASCPC-01 (**A**) and LNCaP/*AR*-sh*RB1*/*TP53* (**B**) NEPC cells in vitro (*n* = 4 biological replicates/group). (**C** and **D**) SCH58261 exerts no inhibitory effect on the proliferation of VCaP (**C**) cells and LNCaP/*AR* (**D**) ADPC cells in vitro (*n* = 4 biological replicates/group). (**E** and **F**) The in vivo tumor growth curves (**E**) and the endpoint tumors (**F**) derived from TRAMP-C1 (TC1) cells that were treated with either vehicle and SCH58261 (vehicle, *n* = 10; SCH58261, *n* = 6; Cells were subcutaneously injected into 6-week-old male BALB/c nude hosts). (**G** and **H**) The in vivo tumor growth curves (**G**) and the endpoint tumors (**H**) derived from Myc-CaP cells that were treated with either vehicle and SCH58261 (vehicle, *n* = 14; SCH58261, *n* = 14; Cells were s.c. inoculated into 6-week-old male FVB hosts). (**I** and **J**) The in vivo tumor growth curves (**I**) and the endpoint tumors (**J**) derived from LASCPC-01 cells that were treated with either vehicle and SCH58261 (vehicle, *n* = 13; SCH58261, *n* = 13; cells were s.c. injected into 6-week-old male BALB/c nude mice). (**K** and **L**) The in vivo tumor growth curves (**I**) and the endpoint tumors (**J**) derived from NCI-H146 cells that were treated with either vehicle and SCH58261 (vehicle, *n* = 5; SCH58261, *n* = 7; cells were s.c. injected into 6-week-old male BALB/c nude hosts). Student’s *t* test was used in **A**–**D**, **E**, **G**, **I**, and **K**. *P < 0.05, **P < 0.01. The SCH58261 powder was dissolved in 3% DMSO, 10% HS-15, and 87% saline solution. 3 mg/kg SCH58261 was i.p. administered to each mouse every other day.

We then investigated whether ADORA2A can be a potential target for the treatment of NEPC and SCLC. We first tested the effect of a potent ADORA2A inhibitor, SCH58261 (SCH), on the cell proliferation of 2 ADPC cell lines, LNCaP/*AR* and VCaP, and 2 NEPC cell lines, LASCPC-01 and LNCaP/*AR*-sh*RB1*/*TP53*. Using the Cell-Titer-Glo assay, we found that SCH significantly inhibited the cell proliferation of LASCPC-01 (Figure 9A) and LNCaP/*AR*-sh*RB1*/*TP53* cells (Figure 9B). No detectable inhibitory effect of SCH was observed on either LNCaP/*AR* (Figure 9C) cells or VCaP cells (Figure 9D). To extend this observation to the in vivo setting, we performed xenograft experiments on PCa cell lines. Importantly, SCH also exerted strongly inhibitory effects on the in vivo growth of the NEPC-like TRAMP cell line TC-1 (Figure 9, E and F), but did not affect the in vivo growth of the ADPC cell line Myc-CaP cells (Figure 9, G and H). Moreover, SCH also strongly inhibited the in vivo growth of human NEPC LASCPC-01 cell derived–xenografts (Figure 9, I and J), suggesting a selective effect of ADORA2A targeting in NEPC treatment.

We further investigated whether SCH could specifically inhibit NE lung cancer. Indeed, SCH strongly suppressed the cell proliferation of SCLC cell lines NCI-H146 (Supplemental Figure 7A) and NCI-H1688 cells (Supplemental Figure 7B), but did not show a repressive effect on LUAD cell lines A549 (Supplemental Figure 7C) or SPC-A-1 (Supplemental Figure 7D). Similar to the in vivo results from the human NEPC cell line LASCPC-01, the xenograft tumor growth of the human SCLC cell line NCI-H146 was strongly inhibited by the ADORA2A inhibitor SCH (Figure 9, K and L). We next asked whether the NE phenotype of SCLC is also dependent on the ADORA2A/PYCR axis. To this end, we downregulated *ADORA2A* in NCI-H146 and NCI-H1688 cells, and found that both PYCR1 and PYCR2 and NE lineage molecules, such as SYP and NSE, were all decreased compared with scramble shRNA transfected cells (Supplemental Figure 7, E and F). Similarly, the global histone acetylation status including H3K27ac, H3K18ac, and H3K9ac were all increased upon ADORA2A-KD in the SCLC cell lines NCI-H146 and NCI-H1688 (Supplemental Figure 7, G and H).

Collectively, the adenosine/ADORA2A signaling played a similarly essential role in 2 NE tumors, including in NEPC and SCLC. These results suggest that ADORA2A blockade may have a broad therapeutic implication in the clinical management of NEPC and SCLC.

## Discussion

Here we show that ADORA2A is selectively upregulated in 2 exemplary treatment-induced NE cancers, NEPC and SCLC. Activated ADORA2A signaling reprograms the proline metabolism via an ERK/MYC/PYCR1/2 axis, thereby enhancing proline synthesis. The latter promotes histone acetylases SIRT6 and SIRT7-mediated H3 deacetylation, which leads to a global downregulation of H3K27ac, triggers cancer cell lineage plasticity, and confers a NE transcriptional profile upon cancer cells. Genetic ablation of *Adora2a* in GEMMs inhibits the development and progression of NEPC and lung cancer, and, intriguingly, prevents the AD-to-NE phenotypic transition. Pharmacological blockade of ADORA2A exerts a strong antitumor effect in vivo in both NEPC and SCLC. Therefore, our study demonstrates that targeting ADORA2A is an attractive therapeutic approach for NE malignancies.

Our study uncovers a link between increased proline synthesis and altered epigenetic modulation of histones, which acts to orchestrate the AD-to-NE lineage transition. Due to the requirement of massive gene expression changes during the lineage shift, epigenetic alteration has been proposed to be actively involved (40). However, the upstream signals and regulators that trigger epigenetic reprogramming remain to be identified. Our results demonstrate that histone deacetylation via SIRT6/7 is enhanced by the adenosine/ADORA2A-promoted proline synthesis in PCa cells. Using cut & tag assays, we uncovered that this H3K27ac reduction led to a global change in transcriptional output, including a decreased luminal lineage signature, increased stem cell gene transcription, and, in particular, an increased NE lineage attribute. ADORA2A signaling was further found to control the proline synthesis through the ERK pathway and its downstream effector MYC. Therefore, we propose a new mechanism of ADORA2A-mediated metabolic-epigenetic cascade via the ERK/MYC/PYCR/SIRT6/7 axis in promoting lineage plasticity and resistance to targeted therapy in cancer cells.

It is worth emphasizing that we have identified another function of ADORA2A in the metabolic regulation of NE epithelial cancer cells, which extends its functions as a coordinator of neural development in the CNS (14–16) or as an immunosuppressive checkpoint modulator in the immune system (41). Careful IHC analysis of the human NEPC and SCLC samples, which are difficult to obtain, as well as mouse cancer organoids and tissues, demonstrate that ADORA2A is highly expressed in the cancer epithelial cells of NEPC and SCLC. Of note, mouse models in the current study with prostate epithelial or lung specific–knockout of *Adora2a* provide solid evidence that *Adora2a* plays an essential role in driving the development and progression of NE prostate and lung cancer in vivo. Knockdown or inhibition of ADORA2A in NEPC or SCLC cell lines also exerts a profoundly repressive role on cancer cell growth in vitro and in vivo, further revealing the value of ADORA2A as an important drug target. On the other hand, it has recently been reported that suppression of adenosine production or ADORA2A activation in renal and mammary carcinomas enhances T cell and natural killer cell function and suppresses MDSCs (42, 43). Therefore, blockade of ADORA2A not only suppresses the aggressive behavior of NE cancer cells, but may also alleviate the immunosuppressive TME, which together lead to a strong antitumor effect on NE malignancies.

Several recent studies have demonstrated an important role for JAK/STAT and FGF signaling in promoting cell lineage plasticity in PCa (44, 45). Using the *Rb1* and *Trp53* double–knockout organoid in vitro system, the authors found that the activation of JAK/STAT and FGF signaling is required for the transition from a luminal tumor cell phenotype to a multilineage, stem cell–like, and AR-independent state, but not for a fully redifferentiated NE-lineage trait, suggesting that an in vivo microenvironment is required to complete the AD-to-NE lineage transition (44, 45). Here, our study provides new insights into the question of how the environmental cues interact with intracellular signaling events in cancer cells to compel a gain of NE lineage traits. The ATP, released and accumulated from dead or damaged cells after targeted therapy or during unrestricted expansion of tumor tissue, is converted to adenosine by CD39 and CD73, which are expressed by tumor-associated cell components such as MDSCs, endothelial cells, and fibroblasts (41). We show in this study that the extracellular adenosine activates the ADORA2A signaling in PCa cells to promote the AD-to-NE lineage transition, highlighting an important role of the TME in controlling cell lineage plasticity and the development of treatment-induced NEPC.

Treatment-induced NE cancer is a category of highly aggressive malignancies with an extremely dismal prognosis and a lack of effective targeted therapies. Our study demonstrates that ADORA2A not only acts as a driver to initiate the proline metabolic-epigenetic cascade in the development of treatment-induced NEPCs and SCLCs, but also is a promising druggable target in NE malignancies. Pharmacological blockade of ADORA2A holds a great translational value in the clinical management of NEPC and SCLC.

## Methods

### The GEMMs and PCa organoids.

The origin and genotyping of *Pbsn-Cre4*; *Rb1^fl/fl^*; *Trp53^fl/fl^*, and *Pbsn-Cre4*; *Pten^fl/fl^*; *Trp53^fl/fl^* were described previously (39). The *Pbsn-Cre4*; *Pten^fl/fl^*; *Hi*-*Myc* mice were obtained by crossing *Hi*-*Myc* with the *Pbsn-Cre4*; *Pten^fl/fl^* line. All GEMM mice used in this study were commercially obtained from The Jackson laboratory. The *Adora2a^fl/fl^* mouse was a gift from Pingjin Gao (School of Medicine, Shanghai Jiao Tong University, Shanghai, China.). *Pbsn-Cre4*; *Rb1^fl/fl^*; *Trp53^fl/fl^* (DKO) mice and *Adora2a^fl/fl^* mice were crossed to obtain the *Pbsn-Cre4*; *Rb1^fl/fl^*; *Trp53^fl/fl^; Adora2a^fl/fl^* (TKO) line. The generation of the murine PCa organoids from these GEMMs were conducted as we previously reported (39). The male mice of C57BL/6J (6-to-8-week-old, male), BALB/c athymic nude mice (6-to-8-week-old, male), and FVB (6-to-8-week-old, male) mice were purchased from Shanghai SLAC Laboratory Animal Company.

### Cell line and cell culture.

LNCaP, VCaP, Myc-CaP, TRAMP-C1 (TC1), A549, NCI-H146, NCI-H1688, and HEK-293T cell lines were purchased from ATCC. The SPC-A-1 cell line was purchased from Shanghai Cell Bank of Chinese Academy of Sciences. The LASCPC-01 cell line was a gift from Qi Wang (Department of Urology, Ren Ji Hospital, School of Medicine, Shanghai Jiao Tong University, Shanghai, China). LNCaP/*AR* cells were established by infecting LNCaP parental cells with an AR-overexpressing lentivirus. LAPC4 cell line was a gift from Charles L. Sawyers (Memorial Sloan Kettering Cancer Center, New York, USA). LNCaP/*AR*-sh*RB1*/*TP53* cells were derived from LNCaP/*AR* cells infected with a lentivirus carrying shRNAs against *RB1* and *TP53*.

VCaP, Myc-CaP, TC1, and HEK-293T were cultured in DMEM medium supplemented with 10% FBS (Gibco) and 0.5% Penicillin Streptomycin (P/S, Gibco). LNCaP, A549, SPC-A-1, NCI-H146, and NCI-H1688 cells were cultured in RPMI-1640 medium with 10% FBS (Gibco) and 0.5% P/S (Gibco). LASCPC1-01 cells were cultured in RPMI-1640 supplemented with 5% FBS, 0.5% P/S (Gibco), 0.005 mg/mL Insulin (Sigma-Aldrich), 0.01 mg/mL Transferrin (Sigma-Aldrich), 30 nM Sodium selenite (Sigma-Aldrich), 10 nM Hydrocortisone (Sigma-Aldrich), 10 nM β-estradiol (Sigma-Aldrich), and 2 mM L-glutamine (Sigma-Aldrich). These cell lines were validated using short tandem repeat (STR) analysis by Shanghai Biowing Applied Biotechnology and were cultured in incubators containing 5% CO_2_ at 37°C.

Other Methods and Materials were presented in the Supplemental Files. All shRNA sequences in this study are incorporated into Supplemental Table 2. RT-qPCR primer sequences are available in Supplemental Table 3. Antibodies used in this study are listed in Supplemental Table 4. The clinical information of PCa samples is presented in Supplemental Table 5.

### Statistics.

All statistical analyses were performed using GraphPad 8.0 software. All data were presented as mean ± SEM. *P* < 0.05 was considered as statistically significant. In brief, the patient survival was analyzed by the Kaplan-Meier analysis, and curves among groups were compared using a log-rank test. The correlation between 2 protein expression levels was analyzed via the Pearson correlation test. 2-tailed student’s *t* tests or Mann-Whitney tests were performed between 2 groups. Kruskal-Wallis test or 1-way ANOVA was used for 3 or more groups. 2-way ANOVA was applied for 2 factors among different groups. Dunnett’s, Tukey’s, or Bonferroni’s posthoc analysis was applied, when applicable, to correct for multiple comparisons.

### Study approval.

All animal experiments performed abide by the regulations of the Laboratory Animal Use and Care Committee Guidelines at Ren Ji Hospital, School of Medicine, Shanghai Jiao Tong University. The animal experiment protocol was approved by the Laboratory Animal Use and Care Committee Guidelines at Ren Ji Hospital, School of Medicine, Shanghai Jiao Tong University. All clinical samples were obtained from Renji Hospital abide by the national ethical regulations and were approved by approved by the Renji Hospital Ethics Committee. All surgical and experimental procedures were approved by the Ethics Committee of Ren Ji Hospital, School of Medicine, Shanghai Jiao Tong University.

### Data availability.

RNA-Seq, ATAC-Seq and cut & tag data in this study have been deposited to the National Genomics Data Center, China National Center for Bioinformation with Bioproject number PRJCA013522. Values for all data points in graphs are reported in the Supporting Data Values file.

## Author contributions

NJ and KZ performed most of the experiments. XC, JW, and PX conducted the RNA-Seq, ATAC-seq, ChIP-Seq, and cut & tag data analysis. KL, LX, CC, and DW assisted the in vivo experiments. HZ, YH, ZJ, ZX, and YS assisted in cell line establishment. WZ, PM, YG, LF, YJ, GZ, and BD helped to collect clinical samples. WB and YZ assisted in vector construction. QW and PZ provided cell lines. WX provided patient information; HHZ, KZ, and NJ interpreted the data. HHZ, KZ, NJ, and WG wrote the manuscript. HHZ, KZ, and WG conceived and supervised this study. KZ, WG, and HHZ funded the project.

## Supplementary Material

Supplemental data

Supporting data values

## Figures and Tables

**Table 1:**
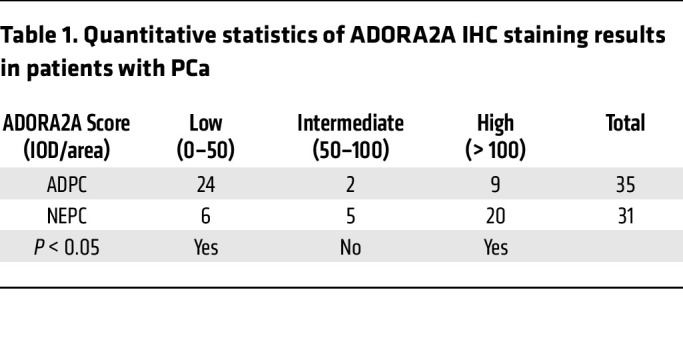
Quantitative statistics of ADORA2A IHC staining results in patients with PCa

## Version 1 12/15/2023

Electronic publication
