# Supplementary material for: ADORA2A-driven proline synthesis triggers epigenetic reprogramming in neuroendocrine prostate and lung cancers
Source: J Clin Invest. 2023 Dec 15;133(24):e168670. doi: 10.1172/JCI168670 (PMC10721152; doi:10.1172/JCI168670)
Supplement: Supplemental data [file jci-133-168670-s160.pdf]

ADORA2A-driven proline synthesis triggers epigenetic reprogramming in neuroendocrine prostate and lung cancers

Na Jing[1,2#], Kai Zhang[1#]*, Xinyu Chen[1], Kaiyuan Liu[1], Jinming Wang[1], Lingling Xiao[3], Wentian Zhang[4], Pengfei Ma[1], Penghui Xu[1,2], Chaping Cheng[1], Deng Wang[1,2], Huifang Zhao[1], Yuman He[1], Zhongzhong Ji[1], Zhixiang Xin[1], Yujiao Sun[1], Yingchao Zhang[1], Wei Bao[1], Yiming Gong[1], Liancheng Fan[1], Yiyi Ji[1], Guanglei Zhuang[1, 5], Qi Wang[1], Baijun Dong[1], Pengcheng Zhang[6], Wei-Xue[1], Wei-Qiang Gao[1,2]*, and Helen He Zhu[1]*

Supplemental material

Supplemental Figures

Supplemental Figure 1

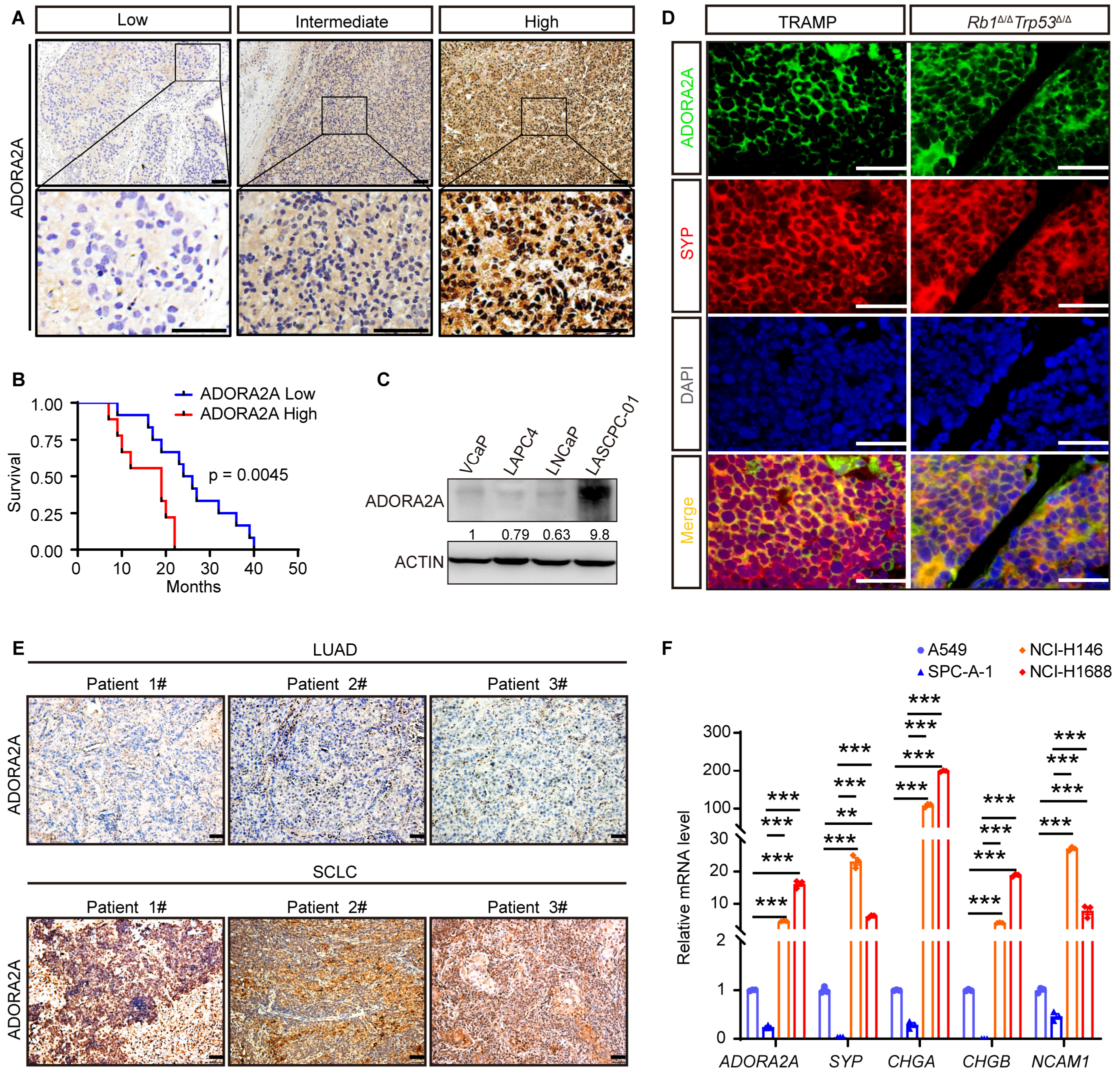

ADORA2A is upregulated in both NEPC and SCLC. Related to Figure 1.

(A) IHC images showing the low, intermediate, and high levels of ADORA2A on our in-house clinical PCa tumor sections. Based on the IHC intensity of ADORA2A, we categorized ADORA2A score into the low (0~50), intermediate (50~100), and high (> 100) levels using the Image J software by calculating IOD/area. Scale bar, 100 µm.

(B) The Kaplan-Meier survival curves of PCa patients with low (n = 12) and high (n = 9) levels of ADORA2A expression.

(C) Immunoblotting result revealing the ADORA2A expression levels in PCa cell lines including VCaP, LAPC4, LNCaP, and LASCPC-01.

(D) IF staining images reveal the co-localization of ADORA2A and SYP in TRAMP

and *Rb1*$^{\Delta/\Delta}$*Trp53*$^{\Delta/\Delta}$ prostate tumor sections. Scale bars, 50 μm.

(E) IHC staining images of ADORA2A in LUAD (n = 14, upper panel) and SCLC (n = 19, lower panel) clinical tumor sections. Scale bars, 100 μm.

(F)RT-qPCR analysis of *ADORA2A* and NE-lineage genes including *SYP*, *CHGA*, *CHGB*, and *NCAM1* in LUAD cell lines of A549 and SPC-A-1, and SCLC cell lines of NCI-H146 and NCI-H1688, respectively (n = 3, biological replicates).

(For statistical analysis, Log-rank test was used in **(B)**; One-way ANOVA with Turkey's post-hoc test was applied for **(F)**. *P < 0.05, **P < 0.01, data are presented as means ± SEM.)

Supplemental Figure 2

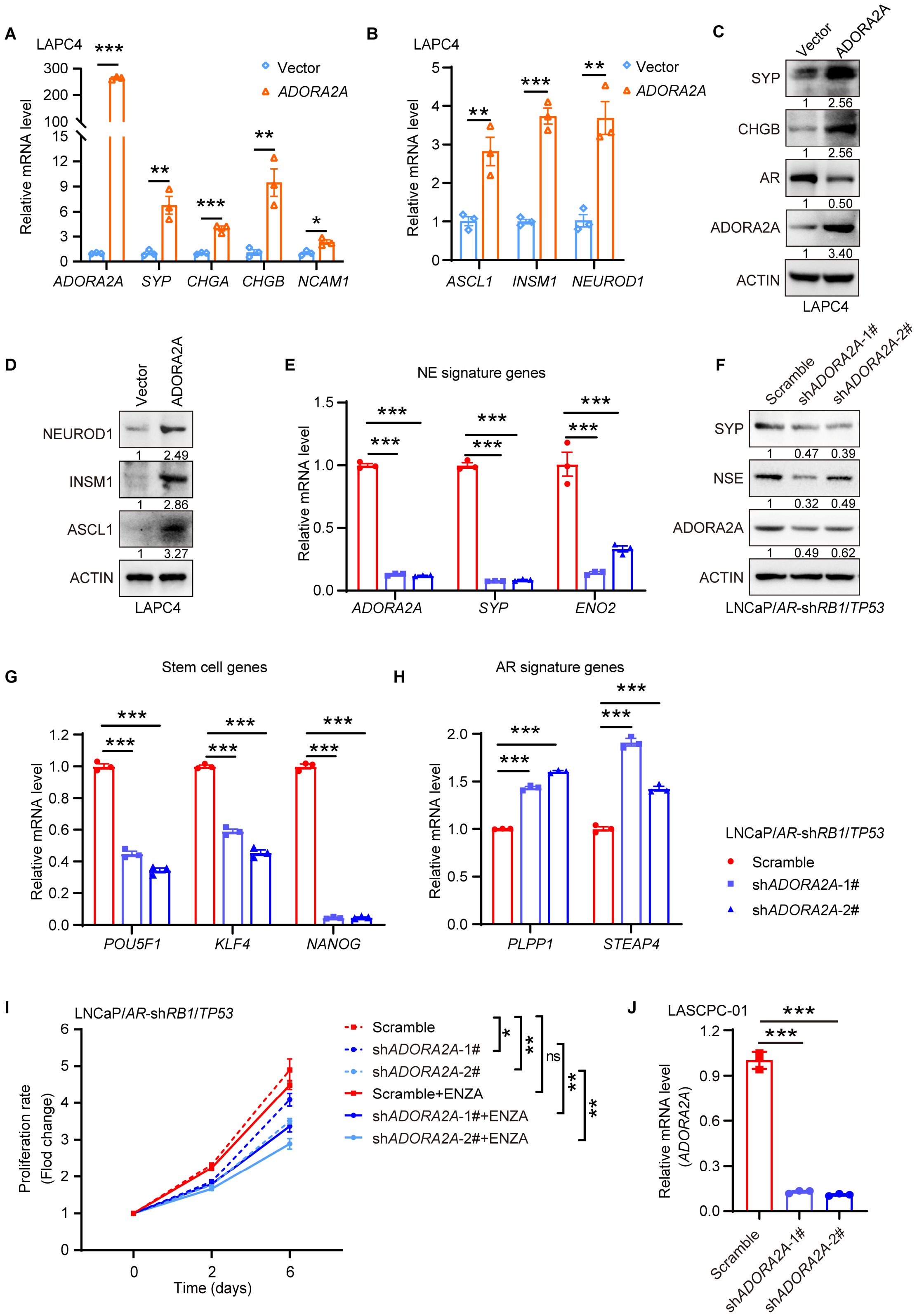

ADORA2A promotes neuroendocrine differentiation and castration resistance in PCa. Related to Figure 2.

(A) RT-qPCR results confirm the ectopic expression of *ADORA2A* and NE-associated genes in LAPC4 cells (n = 3).

(B-D) RT-qPCR **(B)** and immunoblotting **(C, D)** analysis of NE-lineage transcription factors, NE-lineage marker genes, and AR in LAPC4-vector and LAPC4-ADORA2A-OE cells respectively (n = 3).

(E-F) RT-qPCR **(E)** and Immunoblotting **(F)** results validate the knockdown efficiency of ADORA2A and the expression levels of NE-lineage molecules in LNCaP/*AR*-sh*RB1*/*TP53*-Scramble and sh*ADORA2A* cells (n = 3).

(G) RT-qPCR data demonstrate the downregulated stem cell marker genes in LNCaP/*AR*-sh*RB1/TP53* cells upon ADORA2A-KD (n = 3).

(H) RT-qPCR analysis of AR-target genes in LNCaP/*AR*-sh*RB1/TP53* cells upon ADORA2A-KD (n = 3).

(I) The cell growth of LNCaP/*AR*-sh*RB1/shTP53/shADORA2A* and Scramble cells cultured in control medium and enzalutamide (ENZA, 10 μM)-containing medium, respectively (n = 7, biological replicates).

(J) RT-qPCR results validate the knockdown efficiency of *ADORA2A* in LASCPC-01 cells (n = 3).

(For statistical analysis, student's *t*-test was used in **(A** and **B);** One-way ANOVA with Dunnett's post-hoc test was applied for **(E, G**, **H** and **J)**, and Two-way ANOVA with Tukey's post-hoc test was utilized in **(I)**. *P < 0.05, **P < 0.01, ***P < 0.001, ns, non-significant, data are presented as means ± SEM. For RT-qPCR and immunoblotting were repeated three independent experiments, with similar results, and representative images are shown.)

Supplemental Figure 3

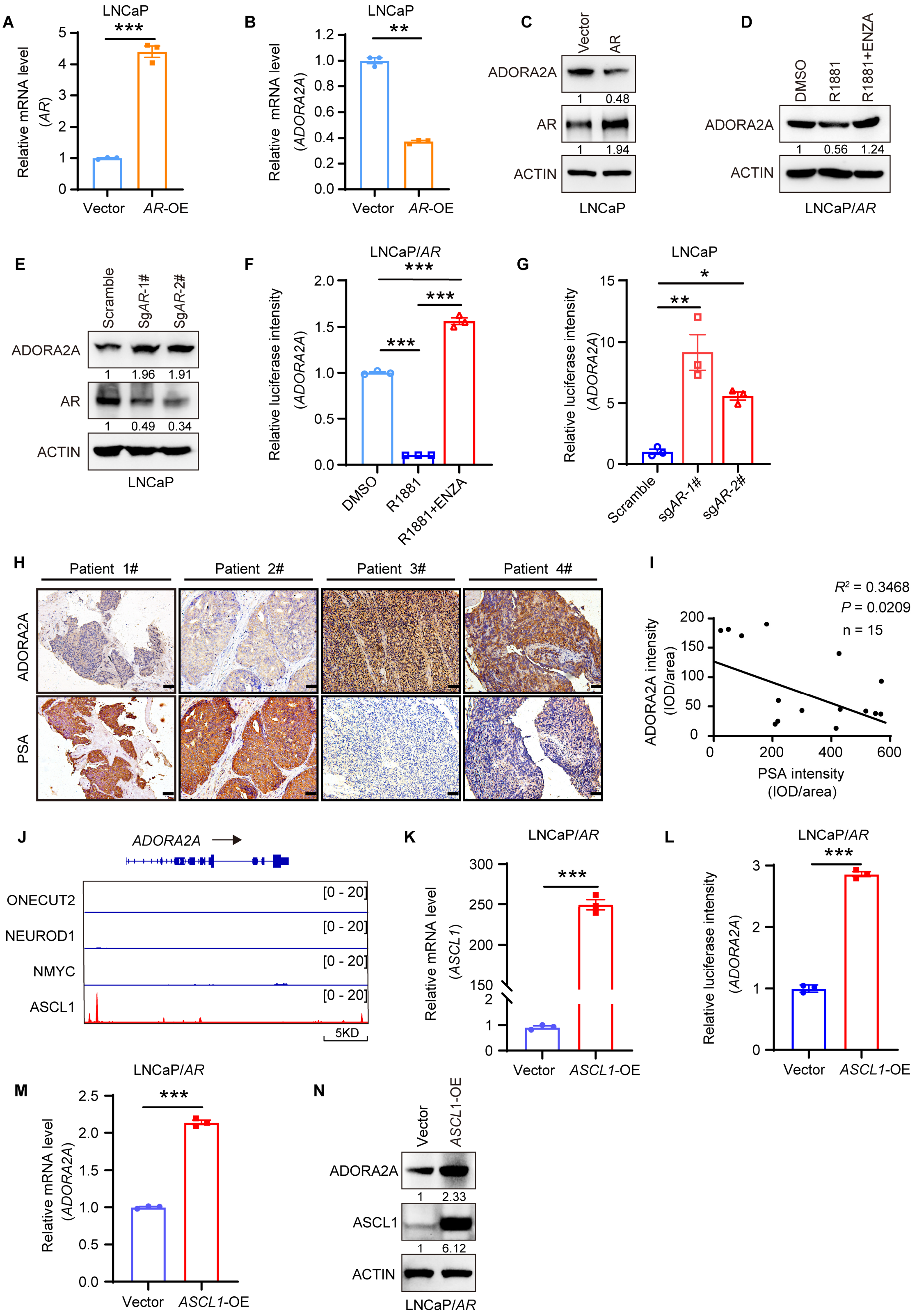

ADORA2A is suppressed by the AR signaling and is activated by ASCL1 in PCa cells. Related to Figure 2.

(A-C) RT-qPCR **(A, B)** (n = 3) and immunoblotting **(C)** results show the levels of AR and ADORA2A in LNCaP/*AR* cells and control cells.

(D) Immunoblots of ADORA2A levels in response to AR agonist R1881 (1 nM), and enzalutamide (10 μM) in LNCaP/*AR* cells treated for 48 hours.

(E) Immunoblotting data showing ADORA2A protein levels in LNCaP-sg*AR* cells and Scramble cells, respectively.

(F) Luciferase assay shows transcription activity of *ADORA2A* upon the stimulation of R1881 or the simultaneous stimulation of R1881 and enzalutamide in LNCaP/*AR* cells (n = 3, biological replicates).

(G) Luciferase assay shows the *ADORA2A* transcriptional activity in LNCaP-sgAR cells and Scramble cells (n = 3, biological replicates).

(H) Representative IHC images show the ADORA2A and PSA levels in human PCa sections. Scale bars, 100 μm.

(I) The IHC staining intensity demonstrates a inversed correlation between PSA and ADORA2A in human PCa sections (n = 15).

(J) ASCL1 shows evident binding peaks on the promoter region of *ADORA2A* gene locus in PCa cells based on the data from Cistrome Data Browser.

(K) RT-qPCR result validates the ectopic expression of *ASCL1* in LNCaP/*AR* cells (n = 3).

(L) Luciferase assay showing the *ADORA2A* transcriptional activity in LNCaP/*AR-ASCL1* cells and control cells (n = 3, biological replicates).

(M-N) RT-qPCR **(M)** (n = 3) and immunoblotting **(N)** results reveal the level of ADORA2A in LNCaP/*AR-ASCL1* and control cells.

(For statistical analysis, student's *t*-test was used in **(A**, **B** and **K-M),** one-way ANOVA with Tukey's post-hoc test was employed in **(F)**, one-way ANOVA with Dunnett's post-hoc test was utilized in **(G)**. $^{*}P < 0.05$, $^{**}P < 0.01$, $^{***}P < 0.001$, data are presented as means ± SEM. For RT-qPCR and immunoblotting were repeated at least three independent experiments.)

Supplemental Figure 4

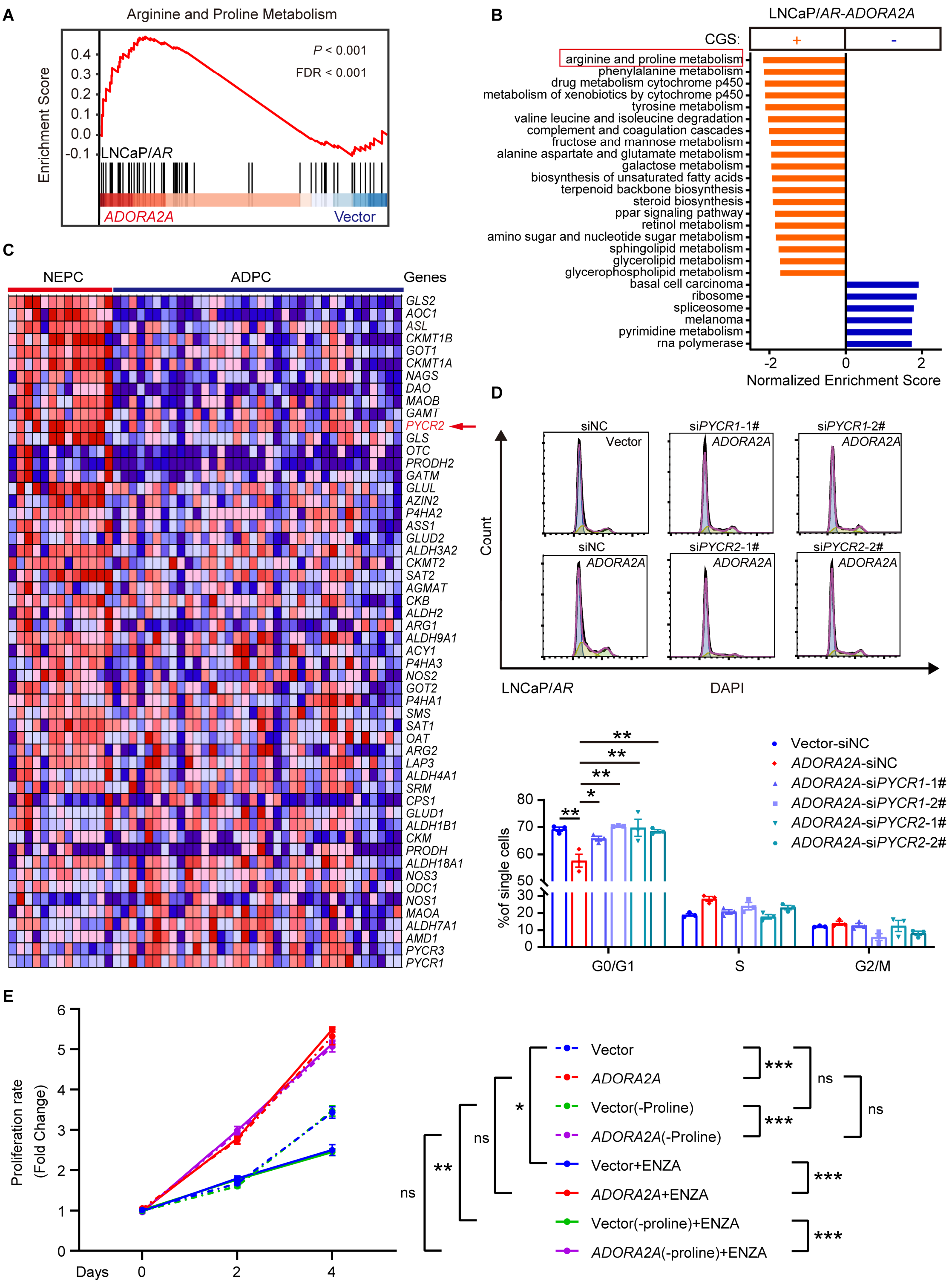

Proline synthesis enzymes are upregulated in NEPC and ADORA2A overexpressing PCa cells. Related to Figure 3.

(A) The GSEA plot shows that the arginine and proline metabolic related genes are enriched in LNCaP/*AR-ADORA2A* cells versus LNCaP/*AR*-vector cells (n = 3,

biological replicates).

(B) GSEA analysis reveals significantly upregulated biological processes and pathways in KEGG enrichment analysis in CGS-stimulated LNCaP/*AR-ADORA2A* cells versus vehicle-treated counterparts (n = 3, biological replicates).

(C) Heatmap showing that *PYCR2*, a key proline synthase in the last step of proline biosynthesis, was among the most upregulated proline metabolic genes in NEPC versus ADPC based on the Beltran PCa data base (1).

(D) Percentage of cells in G_0/G_1, S and G_2/M phases were determined by flow cytometry via DAPI staining. Downregulation of *PYCR1* or *PYCR2* leads to a repressed cell cycle progression in LNCaP/*AR-ADORA2A* cells in CGS-containing medium (n = 3, biological replicates per cell lines).

(E) CCK-8 assay shows the cell growth and sensitivity to enzalutamide of LNCaP/*AR-ADORA2A* and LNCaP/*AR*-vector cells in proline-containing and proline-free medium (n = 6, biological replicates).

(For statistical analysis, one-way ANOVA with Tukey's post-hoc test was used in **(D)**, two-way ANOVA with Tukey's post-hoc test was applied for **(E)**. *$P < 0.05$, **$P < 0.01$, ***$P < 0.001$, data are presented as means ± SEM.)

Supplemental Figure 5

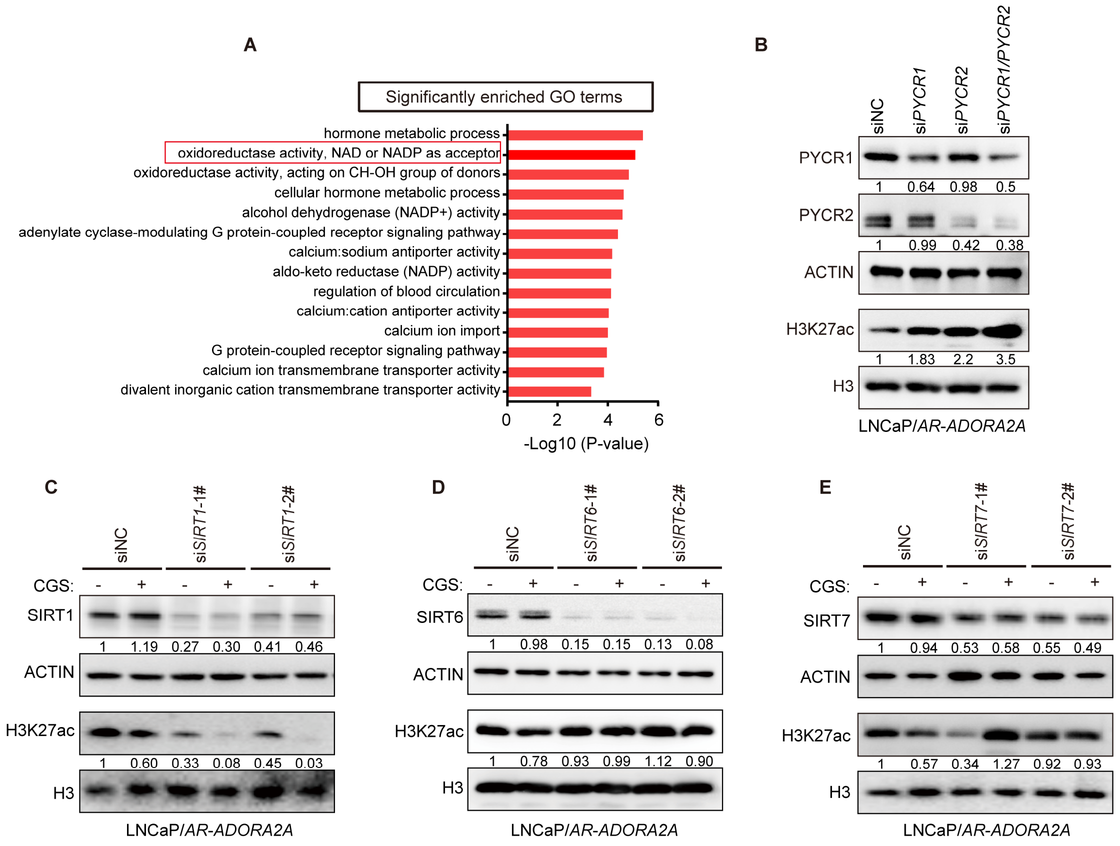

SIRT6 and SIRT7 synergistically suppress H3K27ac upon the activation of ADORA2A signaling in PCa cells. Related to Figure 5.

(A) The GO analysis showing the significantly upregulated signaling pathways in CGS-stimulated LNCaP/*AR-ADORA2A* cells versus vehicle-treated counterparts (n = 3, biological replicates).

(B) Immunoblotting results confirm the synergistic effect of PYCR1 and PYCR2 in modulating H3K27ac status in LNCaP/*AR-ADORA2A* cells.

(C-E) Immunoblotting results demonstrate that the reduced H3K27ac levels were not affected by individual knockdown of SIRT1 **(C)**, but slightly restored by downregulation of either SIRT6 **(D)** or SIRT7 **(E)** in CGS-stimulated LNCaP/*AR-ADORA2A* cells.

Supplemental Figure 6

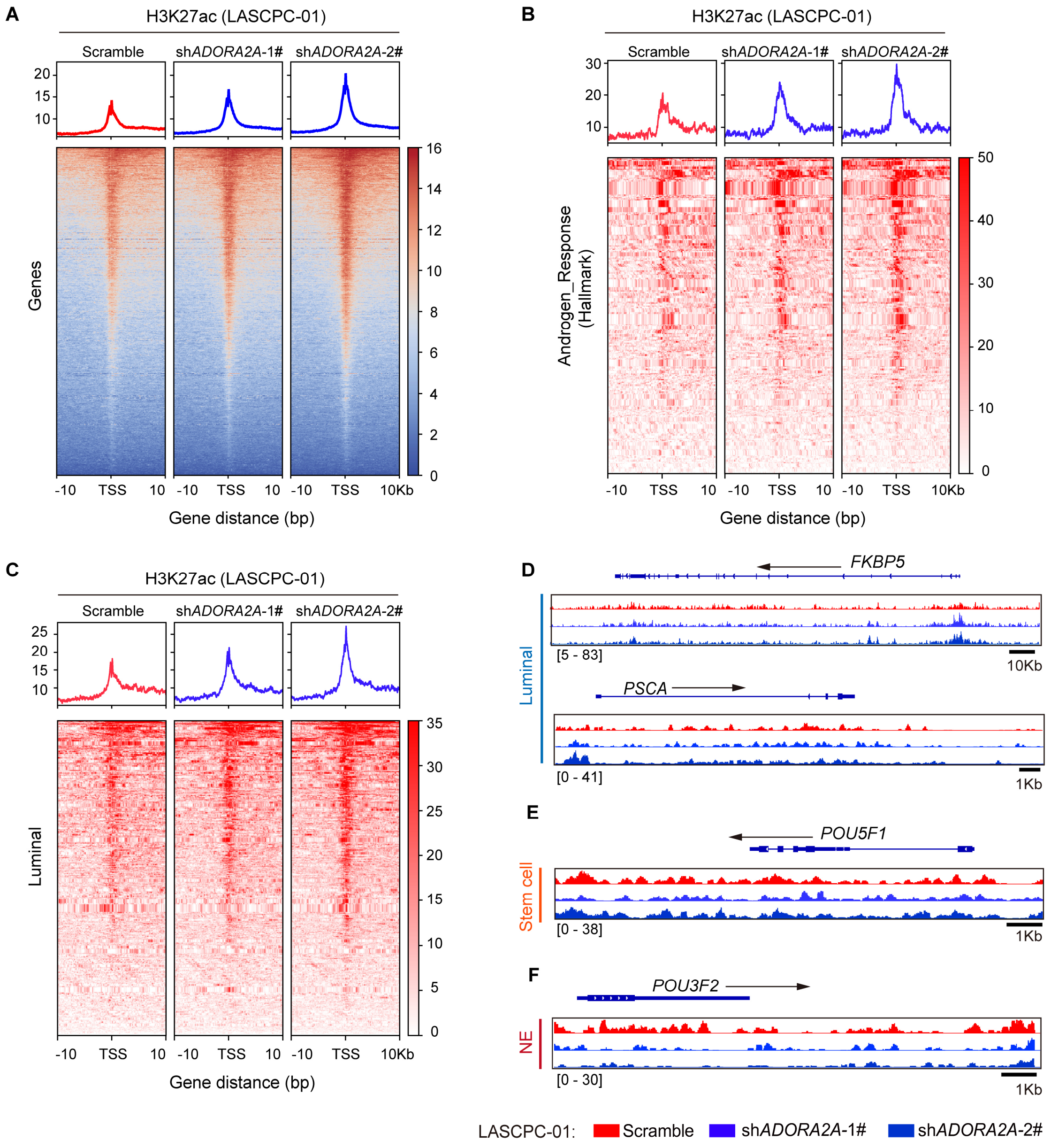

Knockdown of ADORA2A alters global H3K27ac status and affects lineage gene expression in PCa cells. Related to Figure 5.

(A) Cut & Tag data exhibit a globally increased H3K27ac modification in LASCPC-01 cells upon downregulation of *ADORA2A*.

(B-C) Cut & Tag results show that H3K27ac marks of the androgen responsive genes **(B)** and luminal cell marker genes **(C)** are increased in LASCPC-01 cells in response to knockdown of *ADORA2A*.

(D-F) Cut & Tag results exhibit that luminal cell marker genes **(D)** including *FKBP5*

and *PSCA* display increased H3K27ac marks but decreased stem cell gene *POU5F1* **(E)** and neuronal transcription factor *POU3F2* **(F)** in LASCPC-01-sh*ADORA2A* versus LASCPC-01-Scramble cells. For Cut & Tag, n = 2, independent experiments.

Supplemental Figure 7

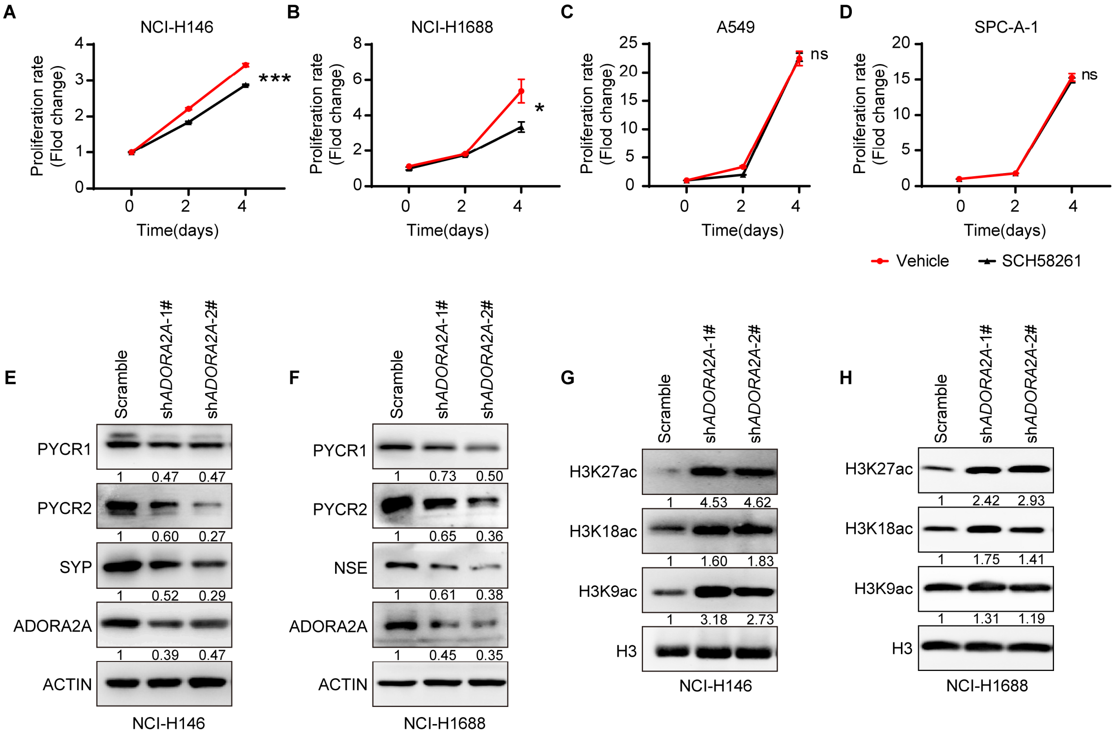

Inhibition or knockdown of ADORA2A suppresses cell proliferation and NE-lineage signature in SCLC. Related to Figure 8.

(A-B) The Cell Titer Glo assay shows that the ADORA2A antagonist SCH58261 significantly restrains the proliferation of SCLC NCI-H146 (n = 6, biological replicates) **(A)** and NCI-H1688 (n = 5, biological replicates) **(B)** cells in vitro.

(C-D) The SCH58261 exhibits little inhibitory effect on the proliferation of LUAD A549 cells (n = 10, biological replicates) **(C)** or SPC-A-1 cells (n = 10, biological replicates) **(D)** in vitro.

(E-F) Immunoblotting assay reveals the reduction of PYCR1/2, and NE-lineage markers including SYP and NSE upon knockdown of *ADORA2A* in NCI-H146 **(E)** and NCI-H1688 cells **(F)**.

(G-H) Immunoblotting assay demonstrates increases in Histone 3 acetylation modifications including H3K9ac, H3K18ac and H3K27ac in NCI-H146 (**H**) and NCI-H1688 (**I**) upon *ADORA2A* knockdown.

(Two-way ANOVA with Bonferroni's post-hoc test was used in **(A-D)**. *P < 0.05, ***P < 0.001, ns, non-significant data are presented as means ± SEM.)

Supplemental Table

Supplemental Table 1

Quantitative statistics of ADORA2A IHC staining results in lung patients

ADORA2A Score (IOD/area)	Low (0~50)	Intermediate (50~100)	High (>100)	Total
LUAD	5	5	4	14
SCLC	3	7	9	19
P<0.05	Yes	No	Yes	

Supplemental Table 2

shRNA, sgRNA and siRNA sequences

**Target Name**	**Sequence (5' to 3')**
sg*AR*-1	AGCAGCAAGAGACTAGCCCC
	CGGCTTAAGCAGCTGCTCCG
sg*AR*-2	CCTCGGTAGGTCTTGGACGG
	TCTCCCCAAGCCCATCGTAG
sh*RB1*	TGTAAGATCTCCAAAGAAATTCAAGAGATTTCTTTGGAGATCTTACA
sh*TP53*	GACTCCAGTGGTAATCTACTTCAAGAGAGTAGATTACCACTGGAGTC
sh*ADORA2A*-1#	TGCTCATGCTGGGTGTCTATT
sh*ADORA2A*-2#	GTTGGCTTGACCAGTCACGTT
si*PYCR1*-1#	CACCATCCATGCCTTGCAT
si*PYCR1*-1#	CCCACTCCTACTCCAGTAT
si*PYCR2*-1#	GGAAGAGGACCTCATCGAT
si*PYCR2*-1#	CCGCTCTCTGCTCATCAAT
si*SIRT1*-1#	GGAAAUAUAUCCUGGACAATT
si*SIRT1*-2#	GCGGGAAUCCAAAGGAUAATT
si*SIRT6*-1#	GCCAAGUGUAAGACGCAUTT
si*SIRT6*-2#	UCCAUCACGCUGGGUACAUTT
si*SIRT7*-1#	GCCAAAUACUUGGUCGUCUTT
si*SIRT7*-2#	GGATTCCGTTGCCTGACACTGT
si*MYC*-1#	GUGCAGCCGUAUUUCUACUTT
si*MYC*-2#	CCACACAUCAGCACAACUATT

Supplemental Table 3

Primers used in RT-qPCR

Primers	Species	Sequence (5' to 3')
RB1-F	human	CAGAAGGTCTGCCAACACCAAC
RB1-R	human	TTGAGCACACGGTCGCTGTTAC
TP53-F	human	CCTCAGCATCTTATCCGAGTGG
TP53-R	human	TGGATGGTGGTACAGTCAGAGC
AR-F	human	ATCCTCATATGGCCCAGTGTC
AR-R	human	GCTCTCTAAACTTCCCGTGGC
KLK3-F	human	GCATGGGATGGGGATGAAGTAAG
KLK3-R	human	CATCAAATCTGAGGGTTGTCTGGA
PLPP1-F	human	TGGAGCGATGTGTTGACTGGAC
PLPP1-R	human	GCAGAGTTGTATGAGAGTCCTCC
PMEPA1-F	human	CTGAGCCACTACAAGCTGTCTG
PMEPA1-R	human	GGATTCCGTTGCCTGACACTGT
STEAP4-F	human	AGTCAGGAGCACTGGATGCAAG
STEAP4-R	human	CTTTGGCTGCCATGAGTGATCC
ENO2-F	human	AGGTGCAGAGGTCTACCATAC
ENO2-R	human	AGCTCCAAGGCTTCACTGTTC
CHGA-F	human	CGCTGTCCTGGCTCTTCTG
CHGA-R	human	TCACCTCGGTATCCCCTTTATTC
SYP-F	human	TTAGTTGGGGACTACTCCTCG
SYP-R	human	GGCCCTTTGTTATTCTCTCGGTA
CHGB-F	human	ACCAGACAGTCCTGACAGAGGA
CHGB-R	human	TAACAGTGCCCACCGCTCCAAT
NCAM1-F	human	CATCACCTGGAGGACTTCTACC
NCAM1-R	human	CAGTGTACTGGATGCTCTTCAGG
ASCL1-F	human	CCCAAGCAAGTCAAGCGACA
ASCL1-R	human	AAGCCGCTGAAGTTGAGCC
INSM1-F	human	CAACAAGTGCCACCCATCCGAA
INSM1-R	human	TCTCCAAGCGAAGGCACAGTTC
NEUROD1-F	human	TCTCCAAGCGAAGGCACAGTTC
NEUROD1-R	human	GCAAAGCGTCTGAACGAAGGAG
ACTB-F	human	CACCATTGGCAATGAGCGGTTC
ACTB-R	human	AGGTCTTTGCGGATGTCCACGT
ADORA2A-F	human	CATGCTAGGTTGGAACAACTGC

ADORA2A-R	human	AGATCCGCAAATAGACACCCA
PYCR1-F	human	TGCCTTGCATGTGCTGGAGAGT
PYCR1-R	human	GCTTCACCTTGTCCAGGATGGT
PYCR2-F	human	TGCAAGCCAGACACATCGTGGT
PYCR2-R	human	GTGTTGGTCATGCAGCGAATCAC
PYCR3-F	human	GTGGAAGCTCAGCACATACTGG
PYCR3-R	human	CTTGGTGGCAAAGATGACGAGC
POU5F1-F	human	CTTGAATCCCGAATGGAAAGGG
POU5F1-R	human	GTGTATATCCCAGGGTGATCCTC
ALDH1A1-F	human	GCACGCCAGACTTACCTGTC
ALDH1A1-R	human	CCTCCTCAGTTGCAGGATTAAAG
NANOG-F	human	TTTGTGGGCCTGAAGAAAACT
NANOG-R	human	AGGGCTGTCCTGAATAAGCAG
Adora2a-F	mouse	GGTAACGTGCTTGTGTGCTG
Adora2a -R	mouse	ACCAAGCCATTGTACCGGAG

Primers used in ChIP-qPCR (5' to 3')

PYCR1-F	Binding site1	TTAAAGTTTCGAGGGGTCCTCT
PYCR1-R	Binding site1	TCCATCCATGCCACCAATCTG
PYCR1-F	Binding site2	CGGGCTTCTCCAAACTCGATGA
PYCR1-R	Binding site2	TCCACGCAGGGCTTTGTCTT
PYCR2-F	Binding site1	TGGAGCTCACGCCAAGCT
PYCR2-R	Binding site1	AATCAGTGGCCAGGATCTCG
PYCR2-F	Binding site2	TGACCTGAGATGAAGTGAGTC
PYCR2-R	Binding site2	AGAGAAGGTGGAAAGATTGT

Primers used in genotyping (5' to 3')

Pbsn-cre-F	CTGAAGAATGGGACAGGCATTG
Pbsn-cre-R	CATCACTCGTTGCATCGACC
Pten-F	CAAGCACTCTGCGAACTGAG
Pten-R	AAGTTTTTGAAGGCAAGATGC
Trp53-F	GGTTAAACCCAGCTTGACCA
Trp53-R	GGAGGCAGAGACAGTTGGAG
Rb1-F	CTCATGGACTAGGTTAAGTTGTGG
Rb1-R	GCATTTAATTGTCCCCTAATCC
Hi-Myc-F	GCATTGGGCATTGTCCATGCCTA

Hi-Myc-R	AGAAGGGTGTGACCGCAACGTA
TRAMP-F	GCGCTGCTGACTTTCTAAACATAAG
TRAMP-R	GAGCTCACGTTAAGTTTTGATGTGT
Adora2a-F	GGGCAAGATGGGAGTCATT
Adora2a-R	ATTCTGCATCTCCCGAAACC

Supplemental Table 4

Antibodies used in this study

**Protein**	**Brand**	**Catalog No:**	**Clone**	**Dilution**
ADORA2A	Abcam	ab3461	Polyclonal	1:1000 for WB; 1:500 for IHC
ADORA2A	Santa Cruz	sc-32261	7F6-G5-A2	1:500 for WB; 1:100 for IF
PYCR1	Proteintech	13108-1-AP	Polyclonal	1:1000 for WB
PYCR2	Proteintech	17146-1-AP	Polyclonal	1:1000 for WB
H3	CST	4499	D1H2	1:1000 for WB
H3K9ac	CST	9649	C5B11	1:1000 for WB
H3K18ac	CST	13998	D8Z5H	1:1000 for WB
H3K27ac	CST	8173	D5E4	1:1000 for WB 1:100 for Cut & Tag
SIRT1	CST	9475	D1D7	1:1000 for WB
SIRT6	CST	12486	D8D12	1:1000 for WB
SIRT7	CST	5360	D3K5A	1:1000 for WB
ACTIN	Abclonal	AC026	Polyclonal	1:2000 for WB
Myc	CST	9402	Polyclonal	1:1000 for WB
P-ERK	CST	9101	Polyclonal	1:1000 for WB
ERK	CST	4695	monoclonal	1:1000 for WB
P-AKT	CST	4060	D9E	1:1000 for WB
Pan-AKT	CST	4691	C67E7	1:1000 for WB
SYP	Abcam	ab32127	YE269	1:1000 for WB; 1:500 for IHC
SYP	BD	611880	AB_399360	1:500 for IF
NSE	CST	24330S	E2H9X	1:1000 for WB
Alexa Fluor 488 donkey anti-mouse	Life Technologies	A21202	Polyclonal	1:500 for IF
Alexa Fluor 594 donkey anti-rabbit IgG(H+L)	Life Technologies	A21207	Polyclonal	1;500 for IF
AR	Abcam	ab133273	EPR1535(2)	1:1000 for WB; 1:300 for IHC

CK8	Abcam	ab53280	EP1628Y	1:300 for IHC
ASCL1	Abcam	ab211327	EPR19840	1:1000 for WB
NEUROD1	ABclonal	A1147	Polyclonal	1:1000 for WB
INSM1	Santa Cruz	sc-377428	monoclonal	1:200 for WB
Ki67	Abcam	ab15580	Polyclonal	1:500 for IHC
Pan-CK	Abcam	ab7753	C-11	1:300 for IHC

Supplemental Table 5

The Clinical information of PCa samples

Patient ID	biopsy	ADORA2A level	ADORA2A intensity (IOD/area)	PSA intensity (IOD/area)	Histology	Survival (Month, M)
1#	A	low	93	570	CRPC-NE	N/A
	B	intermediate				
	C	high				
2#	A	intermediate	82	N/A	CRPC-NE	14M
3#	A	high	150	N/A	CRPC-Ad	19M
4#	A	high	165	N/A	CRPC-Ad	12M
5#	A	low	28	N/A	CRPC-Ad	16M
6#	A	low	19	N/A	CRPC-Ad	27M
7#	A	low	23	N/A	CRPC-Ad	32M
8#	A	low	44	N/A	CRPC-Ad	36M
9#	A	intermediate	60	220	CRPC-Ad	40M
10#	A	low	42	517	CRPC-Ad	9M
11#	A	high	170	95	CRPC-Ad	9M
12#	A	high	181	50	CRPC-Ad	10M
13#	A	high	116	N/A	CRPC-Ad	22M
14#	A	high	140	426	CRPC-Ad	22M
15#	A	high	190	180	CRPC-Ad	7M
16#	A	high	124	N/A	CRPC-Ad	N/A
17#	A	low	32	N/A	CRPC-Ad	N/A
18#	A	high	130	N/A	CRPC-Ad	N/A
19#	A	low	40	N/A	CRPC-Ad	N/A
20#	A	intermediate	59	N/A	CRPC-NE	N/A
	B	intermediate				
21#	A	low	39	N/A	CRPC-NE	N/A
	B	low				
22#	A	low	37	568	CRPC-Ad	9M
23#	A	intermediate	76	N/A	CRPC-Ad	19M
24#	A	high	138	N/A	CRPC-NE	19M
25#	A	low	25	218	CRPC-Ad	24M
26#	A	low	13	416	CRPC-Ad	23M
27#	A	low	20	209	CRPC-Ad	19M
28#	A	low	19	N/A	CRPC-Ad	N/A
29#	A	low	45	N/A	CRPC-Ad	N/A
30#	A	low	48	N/A	CRPC-Ad	N/A
31#	A	low	12	N/A	CRPC-Ad	N/A
32#	A	low	16	N/A	CRPC-Ad	N/A
33#	A	high	179	24	CRPC-NE	20M
34#	A	low	45	430	CRPC-Ad	23M
35#	A	low	43	300	CRPC-Ad	24M
36#	A	low	41	N/A	CRPC-Ad	26M
37#	A	low	48	N/A	CRPC-Ad	39M
38#	A	low	45	N/A	CRPC-Ad	N/A
39#	A	low	46	N/A	CRPC-Ad	N/A
40#	A	low	36	N/A	CRPC-Ad	N/A
41#	A	high	180	N/A	CRPC-NE	N/A
	B	high				
	C	high				
	E	high				
	F	high				
42#	A	high	167	N/A	CRPC-NE	N/A
	B	high				
	C	high				
	D	high				
	E	low				
43#	A	low	38	550	CRPC-Ad	17M

44#	A	high	190	N/A	CRPC-NE	N/A
45#	A	high	123	N/A	CRPC-NE	N/A
46#	A	high	126	N/A	CRPC-NE	N/A
47#	A	high	152	N/A	CRPC-NE	N/A
48#	A	high	121	N/A	CRPC-NE	N/A
49#	A	high	192	N/A	CRPC-NE	N/A
50#	A	intermediate	68	N/A	CRPC-NE	N/A
51#	A	high	124	N/A	CRPC-NE	N/A
52#	A	low	23	N/A	CRPC-NE	N/A
53#	A	high	178	N/A	CRPC-NE	N/A
54#	A	low	43	N/A	CRPC-NE	N/A

Here is the clinical information of the tumor sections of our in-house PCa patients in the current study. Based on the IHC intensity of ADORA2A and PSA, we categorized ADORA2A score into the low (0~50), intermediate (50~100), and high (> 100) levels using the Image J software by calculating IOD/area. Multiple biopsies from the same patient were labeled as A, B, and C, etc.

(**Ad**: prostate adenocarcinoma; **NE**: neuroendocrine cancer; **CRPC**: castration resistant prostate cancer; **N/A**: not available; **IOD**: integral optical density)

Supplemental Methods

Plasmids

The cDNA fragment of human *ADORA2A* was cloned into the pLenti-GV492 vector (Shanghai Genechem Company) with a 3×Flag Tag. The human *AR*-overexpressing lentiviral vector was commercially obtained from addgene (pLENTI6.3/AR-GC-E2325, 85128#). The human ASCL1-overexpressing lentiviral vector was commercially obtained from Miaoling Biology Company (pLV2-CMV-ASCL1-3×Myc-Puro, P44727#). The shRNA targeting *RB1* and *TP53* was simultaneously cloned into the vector of pLenti-MS2-P65-HSF1-Hygro (addgene, 61426#) vector. The shRNA sequence targeting human *ADORA2A* was cloned into the pLenti-CRISPRV2-puro (Addgene, 98290#) vector. SgRNA targeting AR was integrated into the pLenti-CRISPRV2-puro (Addgene, 98290#) vector. The human *ADORA2A* promoter (chr22: 24,427,021-24,430,253, hg38) was cloned into pLenti-CMV-Nano-Glo dual luciferase reporter vector.

Real time quantitative PCR (RT-qPCR) and ChIP-qPCR assays

Total RNA was extracted using TRIzol reagent (Thermo Fisher) following the manufacturer's protocol. 1 µg of total RNA was reversely transcribed into cDNA using the Hi-script II Q RT-Super Mix kit (Vazyme). qPCR was performed using Cham-Q Universal SYBR qPCR Master Mix (Vazyme). *ACTB* was used as an internal control gene. All data were calculated by the $^{\Delta\Delta}$Ct method and performed in triplicates. LNCaP/*AR-ADORA2A* cells were stimulated by CGS21680 (MedChemExpress, HY-

13201, 100 nM) for 48 hours were harvested for ChIP experiments, and DMSO stimulated cells were used as controls. The experiments were conducted using the Simple ChIP Enzymatic Chromatin IP Kit (Magnetic Beads; CST, 9003#) according to manufacturer's protocol. The *PYCR1* and *PYCR2* promoter sequences were analyzed using JASPAR (http://jaspar.genereg.net/) software in search of MYC binding sites. BLAST (blast.ncbi.nlm.nih.gov/Blast.cgi) was employed to design ChIP-qPCR primers for MYC enrichment analysis. All shRNA, siRNA, and sgRNA sequences in this study were listed in Supplemental Table 2. Primers for RT-qPCR and ChIP-seq in the study are listed in Supplemental Table 3.

Immunoblotting assay

Immunoblotting experiments were performed using conventional methods (2). Briefly, cells were lysed using RIPA lysis buffer (10mM Tris-HCl, pH = 8.0, 1mM EDTA, 0.5mM EGTA, 1% Triton X-100, 0.1% Sodium Deoxycholate, 0.1% SDS, 140mM NaCl) supplemented with Protease Inhibitor Cocktail. The protein samples were separated by electrophoresis and the levels of indicated proteins were detected by incubating with primary antibodies and horseradish peroxidase conjugated secondary antibodies. The bands were detected with a chemiluminescence detection system (Bio-Rad). The primary antibodies used in this study are presented in Supplemental Table 4.

H&E, IHC and IF staining assays

H&E, IHC, and IF staining experiments were conducted as we previously reported

(3). Fresh tumor samples were fixed with 10% paraformaldehyde (PFA) overnight at 4°C. These samples were then made into paraffin-embedded blocks. For IHC staining assay, sections were subsequently deparaffinized, rehydrated, and boiled for antigen retrieval (citrate buffer, pH 6.0). Sections were blocked with 10% donkey serum, incubated with primary antibodies overnight at 4°C, and then horseradish peroxidase-conjugated secondary antibody for 1 hour at room temperature. The antigen signal was amplified via diaminobenzidine (DAB)-based chromogenic detection system. For IF staining experiments, sections were incubated with secondary antibodies labeled with Alexa Fluor 488 or 594 florescence (Thermo Fisher Scientific) for 1 hour at room temperature and were counterstained with 4',6-diamidino-2-phenylindole (DAPI). Detailed information of the primary antibodies used for IHC and IF are listed in Supplemental Table 4. Images of H&E and IHC were captured using a microscopic slide scanner (Leica Microsystems). Images of IF staining assays were obtained by a Leica DM2500 microscope (Leica Microsystems).

Luciferase reporter assay

LNCaP/*AR* cells were plated at 40-50% confluency in 24-well plates, and 1 μg of *ADORA2A*-promoter-Dual-Luciferase vector was transfected with Lipo-3000 transfection reagent (Thermo Fisher Scientific). The transfected cells were either stimulated with R1881 alone (10 nM) or concomitantly with R1881 (10 nM) and enzalutamide (10 μM) for 48 hours, and DMSO treated cells served as control. Similarly, LNCaP-sg*AR*, LNCaP-sgScramble, LNCaP/*AR*-vector, and LNCaP/*AR*-*ASCL1* cells were seeded until they grew to 40-50% confluency in 24-well plates. Cells were then

transfected with 1 μg of *ADORA2A*-promoter-Dual-Luciferase vector using Lipo-3000 transfection reagent (Thermo Fisher Scientific). Cells were harvested and lysed using Nano-Glo® Dual-Luciferase® Reporter Assay System kit (promega) 48 hours post transfection. Nano and firefly luciferase activity were measured according to the manufacturer's protocol using a GloMax® 96 Microplate Luminometer (Promega). The nano luciferase activity of each sample was normalized to firefly.

RNA-seq, ATAC-seq, Cut & Tag, and data analyses

These assays were conventionally performed as we previously reported (2). For RNA-seq assay, total RNA was extracted from LNCaP/*AR*-vector and LNCaP/*AR*-*ADORA2A* cells using Qiagen RNeasy kit according to the manufacturer's instructions (Qiagen). Libraries were created using the NEB-Next Ultra TM RNA Library Prep Kit for Illumina (NEB) with index codes added to each sample. The clean reads were then mapped to the human genome (GRCh38/hg38) using Hisat2 (v.2.1.0) to with default settings. Quantification of gene expression was calculated using Stringtie (v.1.3.6). Differentially expressed gene (DEG) analysis was performed with DESeq2. GSEA analysis was performed with the GSEA software 4.2.0. (http://www.gseamsigdb.org/gsea/downloads.jsp) and gene sets were downloaded from MSigdb (http://www.gsea-msigdb.org/gsea/msigdb/search.jsp). Gene Ontology (GO) analysis were conducted using DEG in R (v.4.1.0). Pathways with the following standards including NES value > 1, P value < 0.05, as well as FDR q value < 0.25% were considered significantly enriched. PCa datasets in Beltran cohort (1) and SU2C

cohort (4) were divided into two subgroups (ADPC and NEPC) using NE scores generated by GSVA as previously described (5). For ATAC-seq assay, 2×10^4 organoid cells of *Pten*$^{\Delta/\Delta}$*Trp53*$^{\Delta/\Delta}$ and *Rb1*$^{\Delta/\Delta}$*Trp53*$^{\Delta/\Delta}$ were collected and lysed in 50 µl ice-cold lysis buffer (10 mM pH7.4 Tris-HCl; 10 mM NaCl; 3mM MgCl2; 0.5%NP-40) for 15 minutes on ice. Nuclei fractions were collected to generate sequencing library using TruePrep DNA Library Prep Kit V2 for Illumina (Vazyme, TD501) abide by the manufacturer's instructions. The raw reads were obtained from Illumina Hiseq-PE150 sequencing instrument and aligned to the reference genome (mm9) with Bowtie (v.2.3.5). The sequencing alignment map (SAM) files were eventually converted to BigWig files by SAM tools (v.1.9) and deep Tools (v.3.3.1). The peak visualization was conducted using IGV. We used the ATAC-seq pipeline from Dr. Qu Kun's lab (https://github.com/QuKunLab/ATACpipe). For Cut & Tag experiments and data analysis, we utilized a DNA Binding Profiling Library kit (Yeasen Biotech, 12598ES48#) according to manufacturer's instructions. 1×10^5 cells LNCaP/*AR-ADORA2A* treated with CGS21680 (100 nM, 48 hours) or vehicle were collected for further experiments. The primary antibody of H3K27ac (CST, #8173) was diluted 1: 100 and incubated with the samples overnight at 4 °C and then incubated with the secondary IgG (H+L) antibody (Abcam, ab6702#). pA-Tn5 adapter (0.05 µM) was added to samples for tagmentation, nuclear extraction, and DNA library amplification. The Cut & Tag data analysis was performed using an Illumina Hi-seq-PE150 sequencing instrument. 150-bp paired-end reads were mapped to the genome (GRCh38/hg38) using Bowtie 2 (v.2.3.5). SAMtools (v.1.9) converted Sequence

Alignment/Map (SAM) files to Binary Alignment/Maps (BAM) files. The peak calling and differential peaks were analyzed using SEACR (v.1.3), R (v.4.1.0), and DESeq2 package (v.1.36.0). Heatmaps and plots were generated by deepTools (v.3.3.1). IGV was used for peak visualization. Motif was searched by homer (v.4.11).

RNA-seq, ATAC-seq and Cut & Tag data in this study have been deposited to the National Genomics Data Center, China National Center for Bioinformation with the accession number PRJCA013522.

Xenograft tumor models

For subcutaneous tumor models, 1×10^6 TC1, 2×10^6 NCI-H146 and 2×10^6 LASCPC-01 cells were respectively suspended in 50 µl pre-cold RPMI 1640 medium and mixed with Matrigel (Corning) at a ratio of 1:1 on ice. Then, these cells were subcutaneously inoculated into BALB/c nude mice (6-week-old, male) respectively. Similarly, 1×10^6 Myc-CaP cells mixed with Matrigel at 1: 1 ratio were subcutaneously inoculated into 6-week-old male FVB mice. 10 days after inoculation, we began to monitor tumor size and depict tumor growth curve. The tumor volume was calculated by the following formula: *Tumor volume (mm^3) = (tumor length × tumor height^2)/2*. The mice with comparable tumor burden were divided into two groups and were intraperitoneally administrated with SCH58261 (MedChemExpress, HY-19533, 3 mg/kg, dissolved in 3% DMSO and 10% HS-15 saline) and vehicle every other day. We also measured the body weight of each mouse before they were sacrificed. All mice were euthanized with CO_2 before tumor volume reaches 2000 mm^3.

Cre-expressing adenovirus-driven lung cancer model

The *Rb1*$^{fl/fl}$; *Trp53*$^{fl/fl}$ and *Rb1*$^{fl/fl}$; *Trp53*$^{fl/fl}$; *Adora2a*$^{fl/fl}$ mice (male, 6~8-week-old) were anesthetized and administrated with Cre-expressing adenoviruses (10^8, PFU) via intratracheal injection. Tumor formation can be detectable by histological inspections at around 90 days after Cre-driven depletion of *Rb1* and *Trp53*.

NAD$^+$ measurement, amino acid measurement, and cell proliferation assays

Intracellular amount of NAD$^+$ were measured using NAD/NADH-Glo™ Assay kit (Promega, G9071#) according to the manufacturer's instructions. To determine the intracellular amino acid contents including proline and arginine in LNCaP/*AR*-vector and LNCaP/*AR-ADORA2A* cells stimulated by CGS21680 (100 nM, treated for 48 hours) or/and SCH58261 (25 µM, treated for 48 hours), we utilized a L-8900 automatic amino acid analyzer at the Instrumental Analysis Center of Shanghai Jiao Tong University. The DMSO (vehicle)-stimulated cells served as the control. The cell proliferation assay was performed using cell counting kit-8 (CCK-8, Dojindo, Japan) or Cell-Titer-Glo® 3D cell viability assay (Promega G7570#) according to the manufacturer's instructions. The proline-free medium is prepared by adding 10% dialysed serum (Gibco), 0.5% penicillin/streptomycin (Gibco), L-asparagine (50 mg/L), L-aspartic acid (20 mg/L) and L-glutamic acid (20 mg/L) into DMEM medium. The proline-containing medium is prepared by adding proline (20 mg/L) and hydroxyproline (20 mg/L) into the above-mentioned proline-free medium. The cell proliferation assays were performed with cell counting kit-8 (Dojindo, Japan) or Cell-Titer-Glo® 3D cell viability kit (Promega G7570#) based on the manufacturer's

instructions.

Apoptosis and cell cycle assay

Cell apoptosis was assessed by co-staining of Annexin V-APC (BioGems, 62700-80#) and DAPI (1 μg/ml, Invitrogen, D1306) via flow cytometry. For cell cycle analysis, cells were collected and fixed in 70% ethanol for 4 hours at -20°C, and then stained with 1 μg/ml DAPI for 30 min at 4°C. All flow cytometry assays were performed on the LSR-Fortessa instrument (BD), and data were analyzed using the Flow Jo software.

Human PCa and lung cancer samples

All PCa samples were collected from the Department of Urology, Ren Ji Hospital, School of Medicine, Shanghai Jiao Tong University. According to the diagnostic information provided by the Department of Pathology and Urology at Ren Ji Hospital, these PCa samples were further categorized into ADPC (35 patients, 35 biopsies) and NEPC (19 patients, 31 biopsies) based on IHC results and histopathological analysis. All lung cancer specimens were collected at the Department of Thoracic Surgery, Ren Ji Hospital, School of Medicine, Shanghai Jiao Tong University. These patient tumor samples included LUAD (14 biopsies) and SCLC (19 biopsies). The pathological classification was determined and confirmed by the Department of Pathology of Ren Ji Hospital. Detailed information of the human PCa samples used in this study was presented in Supplemental Table 5.

Reference

1. Beltran H, Prandi D, Mosquera JM, Benelli M, Puca L, Cyrta J, et al. Divergent clonal evolution of castration-resistant neuroendocrine prostate cancer. Nat Med. 2016;22(3):298-305.
2. Cheng C, Wang J, Xu P, Zhang K, Xin Z, Zhao H, et al. Gremlin1 is a therapeutically targetable FGFR1 ligand that regulates lineage plasticity and castration resistance in prostate cancer. Nat Cancer. 2022;3(5):565-80.
3. Zhang K, Guo Y, Wang X, Zhao H, Ji Z, Cheng C, et al. WNT/beta-Catenin Directs Self-Renewal Symmetric Cell Division of hTERT(high) Prostate Cancer Stem Cells. Cancer Res. 2017;77(9):2534-47.
4. Abida W, Cyrta J, Heller G, Prandi D, Armenia J, Coleman I, et al. Genomic correlates of clinical outcome in advanced prostate cancer. Proc Natl Acad Sci U S A. 2019;116(23):11428-36.
5. Su W, Han HH, Wang Y, Zhang B, Zhou B, Cheng Y, et al. The Polycomb Repressor Complex 1 Drives Double-Negative Prostate Cancer Metastasis by Coordinating Stemness and Immune Suppression. Cancer Cell. 2019;36(2):139-55 e10.
